# Role of METTL16 in PPARγ methylation and osteogenic differentiation

**DOI:** 10.1038/s41419-025-07527-x

**Published:** 2025-04-10

**Authors:** Liangjie Lu, Lijun Wang, Minjie Yang, Huihan Wang

**Affiliations:** 1 Department of Orthopedics, Ningbo Medical Center Li Huili Hospital, Li Huili Hospital Affiliated to Ningbo University, Ningbo, China; 2 Department of Pediatrics, The First Hospital of Jilin University, Changchun, China; 3 Department of Orthopaedics, Jiu jiang NO.1 People’s Hospital, Jiu jiang, China; 4 Department of Orthopaedics, Zhengzhou Central Hospital Affiliated to Zhengzhou University, Zhengzhou, China

**Keywords:** Rheumatic diseases, RNA metabolism

## Abstract

Osteoporosis, a prevalent bone disease, is characterized by the deterioration of bone tissue microstructure and imbalanced osteogenesis. The regulatory role of PPARγ m6A methylation mediated by METTL16 remains poorly elucidated. This study utilized advanced single-cell RNA sequencing (scRNA-seq) and Bulk RNA-seq techniques to explore how METTL16 influences the osteogenic differentiation of Bone Marrow-Derived Mesenchymal Stem Cells (BMSCs) and its implication in osteoporosis. The research revealed that METTL16 enhances the suppression of osteogenic differentiation in BMSCs, while PPARγ is associated with BMSC ferroptosis. Mechanistically, METTL16 facilitates the m6A modification of PPARγ transcription, thereby promoting ferroptosis in BMSCs and impeding their osteogenic differentiation. The in vivo animal experiments confirmed the pivotal role of the METTL16-PPARγ axis in osteoporosis development in mice. These findings suggest that the regulation of PPARγ m6A methylation by METTL16, leading to ferroptosis, is a critical mechanism impacting BMSC osteogenic differentiation and the pathogenesis of osteoporosis.

## Introduction

Osteoporosis is a prevalent metabolic bone disease characterized by reduced bone density, deteriorated bone microstructure, and an increased risk of fractures [1–3]. This disease predominantly affects middle and old-age individuals, particularly postmenopausal women [4–7]. In recent years, with the intensification of population aging in China, there has been a steady increase in osteoporosis patients, greatly impacting public health [8]. Therefore, conducting comprehensive research on the pathogenesis of osteoporosis is crucial for effectively preventing and treating the disease [9].

Bone Marrow-Derived Mesenchymal Stem Cells (BMSCs) are recognized as a contributor to the development of osteoporosis, primarily due to their multipotent differentiation potential, particularly their capacity for osteogenic differentiation in diverse cell types [10–12]. Inhibition of the osteogenic differentiation ability of BMSCs could potentially cause a reduction in bone tissue formation, consequently leading to osteoporosis [13]. The ferroptosis-related genes have been demonstrated to inhibit osteogenic differentiation of BMSCs by inducing ferroptosis [14, 15]. Regulating ferroptosis can effectively prevent osteoporosis [16, 17].

In recent years, the significance of m6A methylation, the most prevalent form of RNA modification, has been demonstrated in various biological processes, such as cell fate determination, self-renewal, and differentiation [18–20]. METTL16, a recently identified m6A methyltransferase, plays a vital role in the formation and functioning of m6A [21]. Moreover, METTL16 has been demonstrated to be a risk factor for osteoporosis, significantly upregulated in osteoporotic mice [22]; however, the specific mechanism remains unclear.

We hypothesized that METTL16 may influence ferroptosis in BMSCs by regulating m6A methylation of ferroptosis-related genes, thereby inhibiting their osteogenic differentiation and ultimately leading to osteoporosis. Therefore, this study aims to elucidate this intricate mechanism and the key ferroptosis-related genes regulated by METTL16. We discovered that METTL16 regulates the m6A levels of PPARγ, a nuclear receptor involved in lipid metabolism regulation, thereby promoting the inhibition of PPARγ-mediated ferroptosis and osteogenic differentiation of BMSCs, resulting in osteoporosis. Our findings offer new research directions and therapeutic strategies for future clinical applications.

## Materials and methods

### Construction of osteoporotic model in mice

Female, non-pregnant, SPF-grade C57BL/6JNifdc mice (8 weeks old, n = 219) were purchased from Beijing Vitronichi Experimental Animal Technology Co., Ltd. The mice were housed individually in SPF-grade laboratory conditions with controlled humidity (60–65%) and temperature (25 ± 2 °C). They were provided with ad libitum access to food and water and maintained on a 12-hour light/dark cycle. After a one-week acclimation period during which health conditions were monitored, the experiment commenced. The experimental protocol was approved by the Animal Ethics Committee of our institution [23].

To induce osteoporosis, mice underwent bilateral ovariectomy (OVX) surgery. The procedure included anesthetizing the mice, making a midline incision along the dorsal skin and muscle layer to access and remove the ovaries, and administering penicillin postoperatively to prevent infection. Mice were weighed three days after surgery to monitor recovery. Sham-operated mice underwent similar procedures without ovary removal. After surgery, the OVX and sham groups were housed under SPF conditions (temperature: 25 ± 2 °C, humidity: 50–60%, 12 h light/dark cycle) for four weeks.

The establishment of the osteoporosis model was confirmed using micro-CT scans four weeks post-surgery. Experimental interventions were performed as required, and all mice were euthanized eight weeks after surgery *via* CO₂ inhalation [24].

### H&E staining

Femoral tissue samples were collected, fixed, and sectioned for H&E staining. Sections underwent dewaxing with xylene, followed by sequential dehydration using graded ethanol concentrations (100%, 95%, and 70%). Hematoxylin solution (H8070, Solarbio, Beijing) was applied at room temperature for 5–10 min, followed by rinsing with distilled water and 95% ethanol. Yihong staining solution (G1100, Solarbio, Beijing) was then used for counterstaining for 5–10 min. Dehydration and transparency steps were completed, and the slides were mounted and observed under an optical microscope (Olympus, Tokyo, Japan) [25].

### Micro-CT analysis

Femoral bone growth and structural integrity were assessed using micro-computed tomography (micro-CT) with the mCT-40 system (Scanco Medical, Switzerland). Scans were conducted with the following parameters: 385 μA current, 65 kV voltage, 9 μm pixel size, 1.0 mm AI filter, and 0.4° rotation step. Image reconstruction was performed using Bruker’s NRecon software, and bone morphology was analyzed with CTAn software. A target bone region measuring 0.5 mm × 0.5 mm × 0.25 mm above the femoral head’s growth plate was selected. Trabecular bone was segmented using a constant threshold range of 50–255, with the region of interest defined as the trabecular bone located 1 mm below the distal femoral growth plate.

Micro-CT analysis included several key parameters: bone mineral density (BMD), which indicated the amount and distribution density of bone in the skeletal system; trabecular separation (Tb.Sp), which measured the distance between trabecular fibers to assess bone density and structural integrity; trabecular number (Tb.N), representing the number of intersections between bone and non-bone tissue within a specific length; bone volume (BV), which quantified the total volume of bone tissue and thus the overall bone mass; bone volume to total volume ratio (BV/TV), which showed the proportion of bone surface area relative to total tissue volume; and cortical thickness (Ct.Th) of the femur, which indicated the thickness of the femur’s outer hard layer, critical for bone stability and strength [26, 27].

### Single-cell sequencing and data analysis

Osteoblasts were retrieved using modified dissociation protocols. A randomly selected mouse from the OVX group was modeled and the femoral head was washed three times with Minimum Essential Medium α (MEM α, 12571063, Gibco, USA). The femoral head was then cut into 1–2 mm pieces, and a 10 g fragment was placed into a 50 mL conical tube. Type II collagenase (A004174-0001, Sangon Biotech) dissolved in MEMα with 1% penicillin/streptomycin (15140122, Gibco) was added, and the mixture was gently agitated at 37 °C for 25 min.

After collagenase removal, bone fragments were washed three times with PBS. Following five rounds of digestion, the last two solutions were combined and filtered through a 40 μm filter. The cells were incubated in lysis buffer (R1010, Solarbio, Beijing) for 5 min to lyse red blood cells, then washed twice with PBS. RNA libraries were prepared for sequencing following the standard Illumina protocol [28].

Raw sequencing reads were filtered and disassembled using the PISA software (https://github.com/shiquan/PISA). The filtered readings were mapped to the hg38 genome. PISA was used to generate cell and gene count matrices. The single-cell RNA sequencing (scRNA-seq) data were analyzed using the R software package “Seurat” (version: v4.1.1).

The data underwent a series of quality control measures with filtering thresholds set at nFeature_RNA > 500, nCount_RNA > 1000, nCount_RNA < 20,000, and percent.mt < 5. To mitigate batch effects, the Canonical Correlation Analysis (CCA) method was employed. The data were normalized using the LogNormalize function. Principal Component Analysis (PCA) was then conducted to determine the principal components (PCs) for subsequent t-SNE clustering analysis. Cells were annotated using known lineage-specific marker genes, the online CellMarker database (http://xteam.xbio.top/CellMarker/), and the “SingleR” R package (https://bioconductor.org/packages/devel/bioc/html/SingleR.html). This approach facilitated the identification of marker genes for each cell cluster and enabled subsequent cell annotation. Annotated marker genes corresponding to each cell type are listed in Tables S1 and S2. Temporal analysis was performed using the “monocle” package. The DDRTree algorithm was used for dimensionality reduction, and cell trajectories were inferred based on ranked gene expression trends [29, 30].

### Isolation and culture of BMSCs

Female SPF-grade C57BL/6JNifdc mice, 4 weeks old, were obtained from Beijing VENTOLINHX Experimental Animal Technology Co., Ltd. BMSCs were isolated from bone marrow by flushing the bone marrow cavity with MEM α (12571063, Gibco) and centrifuging at 1000 rpm for 10 min under sterile conditions. The cell pellet was cultured in MEM α with 10% fetal bovine serum (FBS, A4766801, Gibco) and 1% penicillin/streptomycin (PS, 15140122, Gibco) at 37 °C with 5% CO₂. Cells between passages three and six were used for experiments [31].

### Identification of BMSCs and osteogenic differentiation identification

BMSCs were collected using the BD FACS LWA system (BD Biosciences, San Jose, USA) for flow cytometry analysis. The expression of mouse-CD105-FITC (MA5-17945, 0.5 µg/1 × 10^6^ cells), mouse-CD90-PE (A14726, 0.1 µg/1 × 10^6^ cells), mouse-CD73-PE (12-0731-82, 0.125 µg/test), mouse-CD44-FITC (MA5-17,871, 0.2 µg/1 × 10^6^ cells), mouse-CD45-PE (12-0451-82, 0.125 µg/test), and mouse-CD31-FITC (11-0311-82, 1 µg/test) in BMSCs was determined. An isotype control of anti-mouse IgG (31903, 1:500) and a blank control of PBS solution were used. Thermo Fisher Scientific (USA) purchased all the antibodies mentioned above.

BMSCs were cultured at 8 × 10⁴ cells/well in 24-well plates with adipogenic differentiation medium (A1007001, Gibco) for 21 days, changing the medium every 3 days. Oil Red O staining (O1391, Sigma-Aldrich, USA) detected adipocytes. For chondrogenesis, 2 × 10⁵ cells were seeded in chondrogenic medium (A1007101, Gibco) for 21 days, changing the medium every 3 days. Cartilage cells were stained with 1% toluidine blue (89640, Sigma-Aldrich, USA) [32, 33].

BMSCs were also cultured in osteogenic induction medium, supplemented with 10 mM β-glycerophosphate, 100 nM dexamethasone, and 50 mg/mL ascorbic acid (Sigma-Aldrich, USA), with medium changes every 3 days. Alkaline Phosphatase (ALP) staining was performed after 7 days, and Alizarin Red S (ARS) staining after 14 days.

#### ALP staining and assay

ALP staining was performed using the ALP staining kit (40749ES60, Yeasen, China). Cells were fixed with 4% polyformaldehyde for 7 days, washed with PBS, and stained for 30 min. Images were captured with an Olympus IX73 microscope, and Image J was used for quantitative analysis. ALP activity in cell lysates was measured using the ALP activity assay kit (MAK411, Sigma-Aldrich, USA), mixing cell lysates with para-nitrophenyl phosphate and reading absorbance at 520 nm on a Bio-Tek microplate spectrophotometer.

#### ARS staining

To detect calcium deposition in BMSCs after 21 days of induction, ARS staining was performed. BMSCs were fixed with 4% paraformaldehyde and stained with an ARS solution (pH 7.4, PHYGENE, China) at room temperature for 30 min. Following staining, the cells were washed with PBS, visualized under an inverted microscope (IX73, Olympus), and analyzed quantitatively using Image J. For mineralization assessment, the stained calcium deposits were dissolved in a 10% cetylpyridinium chloride (CPC; C0732, Sigma-Aldrich, USA) solution, and absorbance was measured at 562 nm using a microplate spectrophotometer [31, 34].

#### Oil red O lipid staining

Oil Red O staining was used to assess lipid accumulation, following the manufacturer’s instructions (C0157S, Beyotime, China). After PBS washing, the cells were fixed in 4% paraformaldehyde (PFA) for 15 min to prepare for staining [35].

### CRISPR/Cas9

Two METTL16-KO clones, METTL16-KO#1 and METTL16-KO#2, were generated using CRISPR/Cas9 technology. The sgRNA sequences used were as follows: METTL16-sgRNA1: Forward: 5’-GCTTCCAAGAAACAGATACA-3’ (PAM: TGG), Reverse: 5’-GCAAGCAACAAAGAGATACA-3’ (PAM: AGG); METTL16-sgRNA2: Forward: 5’-ATTGCAGTTTGATGAGATGCTG-3’ (PAM: TGG), Reverse: 5’-CCCTTTCCATTCTGACATAAGC-3’ (PAM: AGG). The sgRNAs were integrated into the Lenti-CRISPR v2 vector containing the Streptococcus pyogenes Cas9 gene (HanBio, Shanghai, China). Cells were transduced with the lentiviral Lenti-CRISPR v2 vector to establish METTL16-KO#1 and METTL16-KO#2 cells. Following transfection with sgRNA plasmids and donor sequences, cells were selected using 4 μg/mL puromycin (HY-K1057, MCE, USA). Surviving cells were clonally expanded through limiting dilution, and the METTL16-KO clones were validated via RT-qPCR and Western blot analysis [36, 37].

### 293 T cell culture

The 293 T cell line (ATCC, catalog number: CRL-3216) were cultured in DMEM medium (Catalog number: 11965092, Gibco, USA) supplemented with 10% FBS, 10 μg/mL of streptomycin, and 100 U/mL of penicillin. Cells were cultured in a Heracell™ Vios 160i CR CO_2_ incubator (catalog number: 51033770, Thermo Scientific™, Germany) at 37 °C in a humidified environment with 5% CO_2_. Cells were passaged upon reaching 80–90% confluency [38].

### Construction of shRNA and overexpression lentiviral vectors

Potential short hairpin RNA (shRNA) target sequences were designed based on mouse cDNA sequences retrieved from the GenBank database. Three sequences targeting METTL16 and PPARγ, along with a non-targeting negative control (sh-NC), were synthesized by GenePharma® (Shanghai, China). The lentivirus packaging system was established using the pLKO.1 lentiviral vector for gene silencing and the overexpression lentiviral vector LV-PDGFRA was constructed by Guangzhou RiboBio Co., Ltd.

For lentivirus production, the packaged virus and target vector were transfected into 293 T cells using Lipofectamine 2000 (Thermo Fisher Scientific), achieving 80–90% transfection efficiency. After 48 h, the supernatant containing lentiviral particles was collected, filtered, and centrifuged. Viral load was determined by virus detection assays. Lentiviral vectors overexpressing METTL16 or PPARγ were successfully constructed [39–42].

### Cell transfection

Cells at the logarithmic growth phase were trypsinized, resuspended at a density of 5 × 10⁴ cells/mL, and seeded into 6-well plates at 2 mL per well. Lentiviruses with a multiplicity of infection (MOI) of 10 and a titer of 1 × 10⁸ TU/mL were added to the culture medium. After 48 h, stable cell lines were selected using 2 μg/mL puromycin (UC0E03, Sigma-Aldrich, Germany) for 2 weeks.

The cell transfection groups consisted of the following: (1) sh-NC group, Negative control lentivirus transfection; (2) sh-PPARγ group, Lentivirus targeting PPARγ; (3) sh-METTL16 group, Lentivirus targeting METTL16; (4) oe-NC group, Lentivirus carrying a non-targeting overexpression control; (5) oe-METTL16 group, Lentivirus overexpressing METTL16; (6) oe-PPARγ group, Lentivirus overexpressing PPARγ. The knockdown and overexpression efficiencies were confirmed by RT-qPCR and Western blot analysis 48 h post-transfection.

### RT-qPCR

Total RNA was extracted using Trizol reagent (15596026, Invitrogen, USA) and quantified for concentration and purity at 260/280 nm using a NanoDrop LITE spectrophotometer (ND-LITE-PR, Thermo Scientific™, Germany). Reverse transcription of RNA to cDNA was performed using the PrimeScript RT reagent Kit with gDNA Eraser (RR047Q, TaKaRa, Japan). Quantitative PCR was conducted using SYBR Green PCR Master Mix (4364344, Applied Biosystems, USA) on an ABI PRISM 7500 System (Applied Biosystems).

The primers for each gene were synthesized by the TaKaRa company (Table S4), with GAPDH used as the internal reference gene. To analyze the relative expression levels of each gene using the 2^-ΔΔCt^ method [43–45]. All RT-qPCR detections were performed in triplicate.

### Western blot

Tissues or cells were lysed in RIPA buffer (P0013B, Beyotime Biotechnology, Jiangsu, China) supplemented with protease inhibitors. Protein concentrations were determined using the BCA Protein Assay Kit (P0012, Beyotime Biotechnology). Equal amounts of protein were separated by 10% SDS-PAGE and transferred to a PVDF membrane.

The membranes were blocked with 5% BSA for 2 h at room temperature, followed by incubation with primary antibodies (Table S5) for 1 h at room temperature. After washing, the membranes were incubated with HRP-conjugated secondary antibodies (rabbit anti-goat, ab6721, 1:2000; mouse anti-goat, ab6785, 1:1000, Abcam, UK) for 1 h. Signals were detected using the Pierce™ ECL Western Blot Substrate (32209, Thermo Scientific™, USA), and band intensities were quantified using Image J software. GAPDH served as the internal control. All Western blot experiments were performed in triplicate, and the original images are provided in the supplementary materials [44].

### High-throughput sequencing and analysis of METTL16-KO cells

Total RNA was extracted from bone marrow stromal cells (BMSCs) in the METTL16-WT group (N = 3), METTL16-KO#1 group (N = 3), and METTL16-KO#2 group (N = 3) using the Total RNA Isolation Reagent (12183555, Invitrogen, USA). RNA quality and quantity were assessed via OD measurement, and RNA integrity was evaluated using agarose gel electrophoresis. High-quality total RNA was reverse transcribed into cDNA, followed by RNA library construction and sequencing using Illumina’s NextSeq 500 platform. Base calling converted sequencing data from raw image files into raw reads. Low-quality sequences and adapter contamination were removed using Cutadapt to generate clean reads, which were then aligned to the human reference genome (hg38) using Hisat2. Gene expression levels were quantified using the R software package to generate a gene expression matrix [46].

Differential expression analysis was conducted using the “limma” package in R, with differentially expressed genes (DEGs) identified based on an absolute log fold change (|logFC | ) >5 and an adjusted *p*-value (adj.*P*.Val) < 0.001. Visualization of the results was performed using the ggplot2 (http://had.co.nz/ggplot2/) and pheatmap (https://cran.r-project.org/web/packages/pheatmap/index.html) packages. Enrichment analyses for Gene Ontology (GO) and KEGG pathways were conducted using the SangerBox database (http://sangerbox.com/home.html).

Ferroptosis-related gene data were retrieved from the FerrDb V2 database (http://www.zhounan.org/ferrdb/current/), and Venn diagrams were generated using the Xiantaozi Academic Database (https://www.xiantaozi.com/). Potential m6A target genes of METTL16 and methylation sites of PPARγ were identified using the RM2Target (http://rm2target.canceromics.org/#/home) and SRAMP (http://www.cuilab.cn/sramp) databases.

### Fluorescent dual-luciferase assay

To investigate potential m6A methylation sites, three segments of the PPARγ sequence containing highly reliable m6A sites (Sequence1–3) were inserted into the pmir-GLO dual-luciferase expression vector (General Biosystems, Anhui, China) containing Renilla luciferase (R-luc) and Firefly luciferase (F-luc). This resulted in the construction of dual-luciferase reporter plasmids: pGL3-PPARγ Wild Type and pGL3-PPARγ Mutant. These plasmids, along with control vectors (oe-NC) and METTL16 overexpression vectors (oe-METTL16), were co-transfected into BMSCs. After 24 h of transfection, cells were lysed, and luciferase activity was assessed using the Dual-Luciferase® Reporter Assay System (E1910, Promega).

The steps of the dual-luciferase assay were as follows: 100 μL of Luciferase Reaction Reagent II was added to an Eppendorf tube, then 20 μL of cell lysis solution was carefully aspirated into the tube and mixed by pipetting 2–3 times, F-luc activity was measured using a TECAN Infinite 200 (Tecan Group Ltd., Crailsheim, Germany), 100 μL of stop buffer was added, and R-luc activity was measured, with the ratio of FL/RL used as a measure of relative luciferase activity. Each experiment was repeated three times [47, 48].

### Quantification of total m6A in RNA

The total m6A levels in RNA were quantified using the EpiQuik m6A RNA Methylation Kit (Colorimetric) (P-9005-48, Epigentek, USA). Briefly, total RNA was extracted and quantified using a NanoDrop 2000 spectrophotometer (Thermo Fisher Scientific, USA). For each well, 200 ng of total RNA and binding solution were added and incubated at 37 °C for 1.5 h. Sequentially, capture antibodies, detection antibodies, enhancer solution, color development solution, and stop solution were added, with reactions performed according to the manufacturer’s protocol. Absorbance values at 450 nm were measured using an ELISA microplate reader (Molecular Devices, USA), and m6A levels were compared between groups [49].

### MeRIP-PCR analysis of methylated RNA immunoprecipitation

Methylated RNA immunoprecipitation (MeRIP) was conducted using the Magna MeRIP™ m6A Kit (17-10499, MERCK). A total of 300 μg of RNA was fragmented into ~100-nucleotide fragments and incubated with m6A antibodies (10 μg; ab286164, Abcam). Antibody-bound RNA fragments were captured using Protein A/G magnetic beads (88802, Thermo Fisher Scientific). The samples were incubated for 2 h under rotation, and methylated RNA fragments were subsequently eluted and analyzed by PCR [22].

### Dot blot

RNA was incubated with a minimum of 20× sodium citrate buffer (P4922, Sigma-Aldrich, USA) 95 °C for 5 min. Next, add 100, 200, or 400 ng of Poly(A) RNA to the Hybond N membrane (RPN1520B, GE Healthcare). Following a 30-minute UV crosslinking treatment and blocking with a 5% milk solution, the membrane was incubated overnight 4 °C with an anti-m6A antibody (ab286164, Abcam). Subsequently, the membrane was incubated with secondary antibodies and visualized using enhanced chemiluminescence (ECL) [22]. The relative quantification of Dot blot staining signals was conducted using ImageJ.

### PAR-CLIP

Cells were incubated with 200 mM 4-thiouridine (T4509, Sigma-Aldrich) for 14 h and crosslinked at 365 nm with 0.4 J/cm^2^. After lysis, immunoprecipitation was performed at 4 °C using a METTL16 antibody (ab240595, 1:200, Abcam, Cambridge, UK). The precipitated RNA was labeled with [γ-32-P]-ATP, and radiographic autoradiography was used for visualization. Protein was removed from the PAR fragments by proteinase K digestion, and RNA was extracted for RT-qPCR to detect PPARγ expression levels [50].

### RNA stability analysis

To assess the RNA stability of different groups of BMSCs cells, Actinomycin D (SBR00013, Sigma-Aldrich, USA) was added to the cells at a concentration of 5 μg/ml. After incubating for a specific period, cell samples were collected at 2, 4, 6, and 8 h. RNA was extracted from each cell sample for real-time quantitative polymerase chain reaction (PCR), with GAPDH as the internal reference [51].

### Histological and immunohistochemical analysis

After fixation for 48 h and decalcification for 4 weeks, bone tissue was processed for paraffin embedding and sectioning. Antigen retrieval was conducted by boiling in 0.01 M citrate buffer for 15-20 min. The sections were then incubated at room temperature in 3% H_2_O_2_ for 30 min to inactivate endogenous peroxidase, followed by blocking with goat serum for 20 min at room temperature, with excess liquid removed thereafter. Primary antibodies, METTL16 (ab313743, Abcam, Cambridge, UK) and PPARγ (ab310323, Abcam, Cambridge, UK), were applied, and sections were incubated for 1 h at room temperature. After washing with PBS, an IgG secondary antibody (ab6785, 1:1000, Abcam) was added and incubated at 37 °C for 20 min, followed by PBS washing. Streptavidin-peroxidase was added and incubated at 37 °C for 30 min, then washed with PBS. Sections were developed with DAB (P0202, Beyotime Biotechnology) for 5–10 min, and the reaction was stopped with a 10 min water rinse. Counterstaining was done with hematoxylin (C0107, Beyotime Biotechnology) for 2 min, followed by differentiation with hydrochloric acid alcohol, a 10 min water rinse, and graded alcohol dehydration. Finally, sections were cleared in xylene and mounted with 2–3 drops of neutral resin. Observations and quantifications were performed under an upright microscope [52].

### CCK-8 assay

BMSCs were seeded at a density of 5 × 10^3^ cells per well in a 96-well plate to assess cell viability. Cell viability was determined following the manufacturer’s instructions using the CCK-8 assay kit (catalog number ab228554, Abcam, USA). The cells in each group were cultured in separate wells of a 96-well plate with 2500 cells per well. The cells were cultured for 24, 48, 72, and 96 h. At the designated time points, 10 μL of CCK-8 was added to each well. Absorbance measurements were performed using an enzyme-linked immunosorbent assay (ELISA) reader (Tecan M1000 PRO) at a wavelength of 450 nm [53].

### Scratch assay

The treated test cells were seeded into 6-well plates at a cell density ranging from 70% to 90%. A 200 μL pipette tip was used to create a clear and visible scratch across the well. The cells were washed to remove any detached cells, and fresh culture medium was added. The plates were incubated at 37 °C with 5% CO₂.

The initial scratch condition (0 h) was recorded using an inverted microscope (Olympus CKX53, Japan) for observation and photography. After 24 h, the scratches were observed and photographed again using the same microscope. The scratch width was measured, and the cell migration distance was calculated using ImageJ software [54, 55].

### Transwell experiment

The extracellular matrix gel from Sigma-Aldrich (E1270, Germany) was added to the upper chamber of a 24-well Transwell plate (8 μm) and incubated at 37 °C incubator for 30 min to allow solidification. Cells transfected for 48 h were collected and a suspension of 1 × 10^5^ cells in a serum-free medium was prepared. A total of 200 μL of cell suspension (2 × 10⁴ cells/well) was added to the upper chamber, while 800 μL of medium containing 20% FBS was added to the lower chamber. After incubating at 37 °C for 24 h, the Transwell plate was removed and rinsed twice with PBS. The membranes were fixed in formaldehyde for 10 min, washed with water three times, and stained with 0.1% crystal violet at room temperature for 30 min. Excess stain was removed with PBS, and non-invaded cells on the upper surface were gently wiped off with a cotton swab. Images of the invaded cells were captured using an inverted light microscope (Olympus CKX53, Japan). The number of invaded cells was analyzed using Image J software [54, 56, 57].

### TUNEL staining

Cells were fixed in 4% paraformaldehyde (60536ES60, Shanghai Yeeson Biological Technology Co., Ltd., China) at room temperature for 15 min and permeabilized with 0.25% Triton X-100 for 20 min. Blocking was performed using 5% bovine serum albumin (BSA, Shanghai Yeeson Biotechnology Co., Ltd., China).

Mouse bone tissue samples were embedded in paraffin, sectioned, and treated with 20 mg/mL DNase-free proteinase K (ST532, Bellate BioTech Co., Ltd., Jiangsu) at 20 °C for 15 min. Sections were incubated with 3% hydrogen peroxide for 20 min, followed by three PBS washes. TUNEL reagent (C1086, BCI Biotech Co., Ltd., Shanghai) was applied in the dark, and DAPI staining solution (C1002, Beryllite Biotechnology Co., Ltd., Shanghai) was used for nuclear labeling. Images were captured using a confocal microscope (LSM 880, Carl Zeiss AG, Germany), where apoptotic cells exhibited green fluorescence (TUNEL-positive), and all nuclei were stained blue with DAPI. The apoptosis rate was calculated as the percentage of TUNEL-positive cells [58].

### Detection of intracellular Fe^2+^ content

Intracellular Fe^2+^ levels were measured using the FerroOrange probe (F374, Dojindo, Japan). Preprocessed BMSCs were seeded into confocal culture dishes, washed with Hank’s Balanced Salt Solution (HBSS; 13150016, Gibco), and incubated with 1 μM FerroOrange for 30 min. Observations were made using a confocal laser scanning microscope (LSM780, Zeiss).

Iron levels in cell and tissue lysates were determined using the iron assay kit (ab83366, Abcam) according to the manufacturer’s instructions [59].

### Transmission electron microscopy

BMSCs and bone tissue samples were fixed overnight in 2.5% glutaraldehyde at 4 °C and post-fixed with 1% osmium tetroxide at room temperature for 1–2 h. Samples were dehydrated using graded ethanol concentrations (50%, 70%, 80%, 90%, and 95%), treated with pure acetone, and embedded in resin overnight at 70 °C. Ultrathin sections (70–90 nm) were prepared using a Reichert ultramicrotome and stained with lead citrate and saturated uranyl acetate in 50% ethanol for 15 min each. Sections were observed under a transmission electron microscope [59].

### Mitochondrial membrane potential detection

BMSCs were seeded into 6-well plates and stained using the JC-1 mitochondrial membrane potential assay kit (40706ES60, Yikesan Biotech, Shanghai, China). A total of 1 mL JC-1 staining solution was added to each well and incubated at 37 °C for 20 min. CCCP (50 μM), provided in the kit, was used as a positive control. Following incubation, the cells were washed twice with JC-1 staining buffer (1×) and resuspended in cell culture medium containing serum and phenol red. Fluorescence was observed using a laser confocal microscope with excitation and emission wavelengths of 490 nm and 530 nm, respectively [60].

### Biochemical analysis

Glutathione (GSH) and oxidized glutathione (GSSG) levels were measured using the GSH and GSSG assay kit (S0053, Beyotime Biotechnology Co., Ltd.) according to the manufacturer’s protocol. Absorbance was recorded at 450 nm using a microplate reader (Infinite200, Tecan, Beijing) and quantified using a standard curve.

Reactive oxygen species (ROS) levels were determined using the fluorescent probe DCFH-DA (S0033S, Beyotime Biotechnology Co., Ltd., Jiangsu) with a flow cytometer (FC500ML, Beckman, USA) following staining with 10 μM DCFH-DA for 20 min in the dark. ROS visualization was performed using confocal microscopy (LSM780, Zeiss) at 488 nm excitation and 525 nm emission.

Lipid peroxidation was assessed using the DHE probe (BH-02×9621, Shanghai Bohu Biotechnology Co., Ltd.), with fluorescence intensity measured at 488–535 nm excitation and 610 nm emission. Malondialdehyde (MDA) levels were quantified using the MDA assay kit (BC0025, Solarbio, Beijing) as per the manufacturer’s instructions [59, 61, 62].

### In vivo animal experiments

Lentivirus (1 × 10⁸ plaque-forming units/100 μL) was injected into mice through the tail vein, with two injections separated by at least two days and completed 7 days prior to surgery [63].

The mice were randomly allocated into five groups, each containing six mice, as follows: the Sham group (mice without model induction), the OVX group (mice with OVX model induction), the sh-NC + oe-NC group (mice without knockdown of METTL16 and overexpression of PPARγ), the sh-METTL16 + oe-NC group (mice with knockdown of METTL16 and without overexpression of PPARγ), and the sh-METTL16 + oe-PPARγ group (mice with knockdown of METTL16 and overexpression of PPARγ).

### Statistical analysis

Statistical analyses were conducted using R (version 4.2.1) in the RStudio IDE (version 2022.12.0-353). File preprocessing was performed with Perl (version 5.30.0). Statistical graphs were generated using GraphPad Prism (version 8.0), and violin plots were created with the Hiplot database.

Quantitative data are presented as mean ± standard deviation (SD). Comparisons between two groups were conducted using independent samples t-tests. One-way ANOVA was used for comparisons among multiple groups, while two-way ANOVA was applied for comparisons across time points within groups. Post hoc analyses were performed using Bonferroni correction. Statistical significance was defined as *P* < 0.05 [64, 65].

## Results

### Comprehensive analysis of osteoporosis in OVX mouse model: bone morphology, scRNA-seq, and principal component insights

We created a mouse model of osteoporosis induced by OVX and examined the distal femur of mice from the Sham and OVX groups using H&E staining. Staining results revealed that the cortical bone trabeculae in the distal metaphysis of the femur were thick, well-arranged, and dense in the Sham group mice. Additionally, the structure of the trabeculae was clear and intact. In contrast to the Sham group, the distal femoral metaphysis in the OVX group of mice showed a different pattern (Fig. S1A).

Using Micro-CT, we observed the bone mineral density (BMD), BV/TV, Tb. N, BV, Tb. Sp, and ct.Th of the distal femur in mice. The results revealed that, compared to the control group, mice in the OVX group exhibited thinner trabecular bones characterized by disorganized trabecular structure, reduced connectivity, increased separation, and thinner cortical bones. Moreover, there were decreases in BMD, BV/TV, and Tb. N, BV, and ct.Th, while Tb. Sp showed an increase, thus indicating the successful modeling of the OVX group mice (Fig. S1B-H).

Hence, we chose a mouse bone tissue sample from a model of the OVX group to analyze scRNA-seq. Data analysis was performed using the ‘Seurat’ package in the R software. Following quality control and standardization, Fig. S2A illustrates the resulting distribution of cellular RNA. The correlation coefficient between nCount and percent. Mt is r = -0.12, whereas the correlation coefficient between nCount and nFeature is r = 0.73. These findings suggest a positive correlation between nCount and nFeature, indicating good cell quality after filtration (Fig. S2B). Finally, we identified a set of highly variable genes in the bone tissue of osteoporotic mice from a filtered pool of 1230 cells. To obtain the top 3500 changing genes for downstream analysis (Fig. S2C), we utilized the function ‘FindVariableFeatures’. The results suggest an abundance of highly variable genes in the tissues of individuals with osteoporosis.

PCA was subsequently performed to reduce the dimensionality of the 3500 genes above, thus enabling cells’ t-distributed Stochastic Neighbor Embedding (t-SNE) clustering. Following PCA dimension reduction, we identify the PCs that are considered important based on their low *p*-values. PCA was performed, resulting in 40 PCs (Fig. S2D). The JackStrawPlot function was utilized to visualize each PC and compare the position of the *p*-value distribution with the mean distribution. The analysis revealed that the so-called “important” PCs generally exhibit smaller *p*-values, as indicated by the solid line above the dashed line. These PCs effectively capture the information encoded in the highly variable genes filtered previously (Fig. S2E). This study presents the rankings of the top 7 PCs obtained through PCA for various cell samples. Additionally, we provide the heatmaps depicting the characteristic genes of these top 7 PCs and their respective gene expression levels (Fig. S2F-G).

The results above suggest that the PCA outcomes are considerably reliable. Subsequently, the seven highest-ranked PCs are chosen for t-SNE clustering and cell annotation.

### Single-cell sequencing unveils distinct cell clusters and highlights METTL16’s role in osteoblast differentiation in osteoporotic mice

**Fig. 1:**
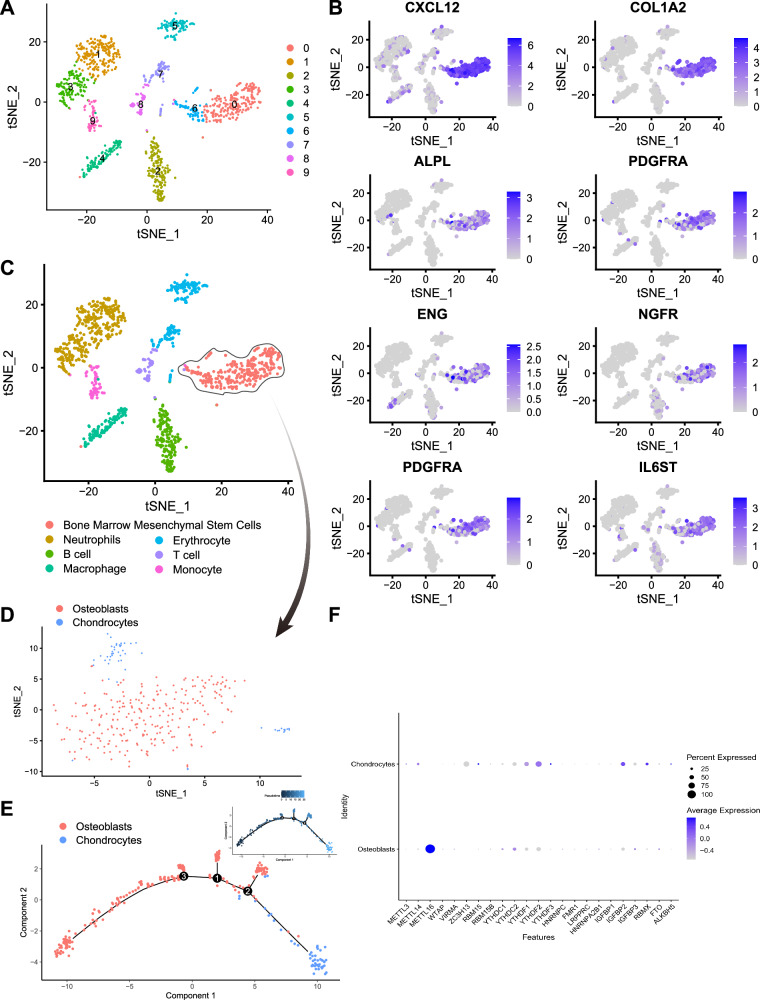
Cell annotation analysis of scRNA-seq. **A** t-SNE analysis results displaying 10 cell clusters; **B** UMAP expression distribution of specific marker genes for Bone Marrow Mesenchymal Stem Cells; **C** Annotation of 10 cell clusters to 7 cell types; **D** t-SNE analysis identifying 2 subclusters within the Bone Marrow Mesenchymal Stem Cells cluster; **E** Pseudotime analysis of subclusters; **F** Dot plot revealing the expression differences of 23 m6A regulators between Osteoblasts and Chondrocytes. The Y-axis shows only the two cell types, with no other significance.

We utilized TSNE clustering analysis to categorize the single-cell sequencing dataset into 10 distinctive cell clusters (Fig. 1A). We annotated these 10 cell clusters using the “SingleR” package in Bioconductor/R and relevant literature and data from the CellMarker online website. Figure S3A presents the heatmap of the top 10 differentially expressed genes, while Fig. S3B displays the scatter plot of the annotated genes.

These ten cell clusters correspond to seven cell types, including neutrophils, B cells, macrophages, red blood cells, bone marrow mesenchymal stem cells, T cells, and monocytes (Fig. 1C).

Specific marker genes, such as CXCL12, COL1A2, ALPL, PDGFRA, and ENG, clearly indicate the presence of bone marrow mesenchymal stem cells (Fig. 1B). By conducting additional analysis on bone marrow mesenchymal stem cells, we identified subpopulations within their internal structure. Figure S4A displays the heatmap of differential genes, while Figure S4B showcases the dot plot of annotated genes. These subgroups mainly comprise two types of cells: osteoblasts and chondrocytes (Fig. 1D).

Temporal analysis revealed a gradual decrease in the number of osteoblasts in the bone tissue of osteoporotic mice over time (Fig. 1E). It is worth noting that among the known m6A regulatory factors, only the METTL16 gene is highly expressed in osteoblasts, while it is nearly absent in chondrocytes (Fig. 1F). This result indicates that the gene likely plays a pivotal role in differentiating mesenchymal stem cells into osteoblasts within the bone marrow.

Initially, METTL16 was believed to be a prominent methyltransferase involved in the methylation process. Subsequently, it was confirmed to impact the progression of specific cancers [21, 66, 67]. Furthermore, previous studies have demonstrated that METTL16 is a risk factor for osteoporosis and exhibits a notable upregulation in osteoporotic mice [22].

Using in-depth analysis of single-cell sequencing data, we have revealed the internal structure and function of mesenchymal stem cells from the bone marrow and identified METTL16 as a crucial gene involved in their differentiation into bone cells.

### METTL16 upregulation in osteoporotic mice and its inhibitory impact on osteogenic differentiation of BMSCs: experimental validation and genetic manipulation insights

**Fig. 2:**
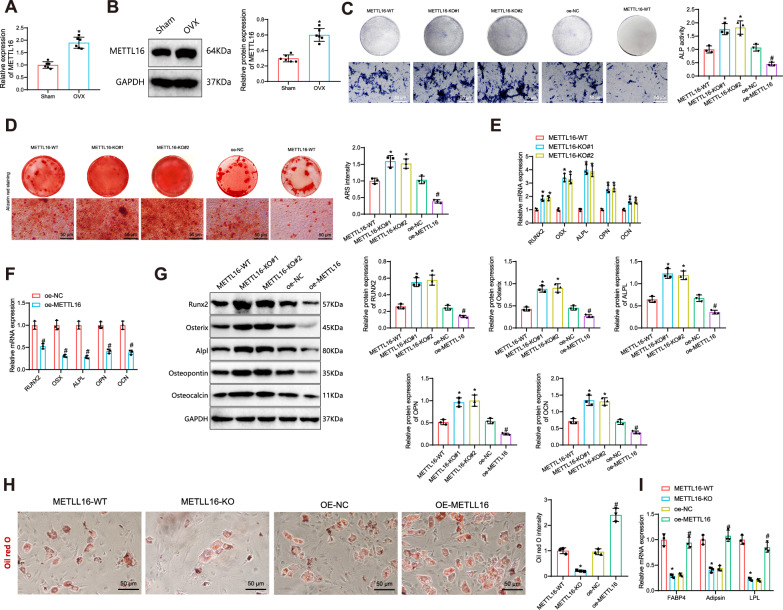
Effect of METTL16 on osteogenic differentiation of BMSCs. **A** Gene expression levels of METTL16 in mouse bone tissues of different groups detected by RT-qPCR; **B** Protein expression levels of METTL16 in mouse bone tissues of different groups detected by Western blot; **C** ALP staining showing the ALP activity of BMSCs in different groups, scale bar: 50 μm; **D** ARS staining assessing the osteogenic differentiation of BMSCs in different groups, scale bar: 50 μm; **E**, **F** Gene expression levels of osteogenic differentiation markers RUNX2, Osterix, ALPL, OPN, and OCN in BMSCs of different groups detected by RT-qPCR; **G** Protein expression levels of osteogenic differentiation markers RUNX2, Osterix, ALPL, OPN, and OCN in BMSCs of different groups detected by Western blot; **H** Oil red staining in BMSCs of each group, scale bar: 50 μm; **I** RT-qPCR to detect the gene expression levels of adipogenic differentiation-related genes in BMSCs of each group. * indicates *p* < 0.05 compared to the METTL16-WT group, # indicates *p* < 0.05 compared to the oe-NC group, cell experiments were repeated three times with 6 mice per group.

Based on the scRNA-seq results, we assessed the expression levels of METTL16 in mouse bone tissue from the Sham and OVX groups using RT-qPCR and Western Blot. The findings revealed an upregulation of METTL16 in osteoporotic mice (Fig. 2A, B), as evidenced by the notably increased protein and mRNA levels of METTL16 in the OVX group compared to the Sham group.

To further validate the impact of METTL16 on the osteogenic differentiation of BMSCs, we isolated BMSCs from mice and induced differentiation into adipocytes, osteoblasts, and chondrocytes to demonstrate their multi-lineage differentiation potential (Fig. S5A). The positive expression of CD44, CD73, CD90, and CD105 was detected in BMSCs by flow cytometry, while CD31 and CD45 were negative (Fig. S5B). This result implies that the isolated cells are indeed BMSCs.

Subsequently, we employed CRISPR/Cas9 gene editing technology to generate METTL16-KO BMSCs, with METTL16-WT as the wild-type control. The expression level of METTL16 in individual clone cells was assessed using RT-qPCR and Western blot techniques. Clones with no detectable METTL16 expression (Fig. S5C-D) were chosen for subsequent expansion in culture. We simultaneously constructed overexpressing METTL16 BMSCs cells using lentiviral vectors, with oe-METTL16 and oe-NC as controls. Subsequently, we validated the transfection efficiency through RT-qPCR and Western blot techniques, as shown in Fig. S5E-F.

The impact of METTL16 knockout or overexpression on the osteogenic differentiation of BMSCs was investigated by conducting ALP staining on the 7th day and ARS staining on the 14th day. The results showed that compared to the METTL16-WT group, there was a significant increase in the intensity of ALP and ARS staining in the METTL16-KO#1 and METTL16-KO#2 groups, indicating that inhibiting METTL16 significantly promotes the osteogenic differentiation of BMSCs. Conversely, compared to the oe-NC group, the osteogenic differentiation level of BMSCs was reduced in the oe-METTL16 group (Fig. 2C, D). Runx2, Osterix (OSX), Alpl, Osteopontin (OPN), and Osteocalcin (OCN) were detected as bone formation markers using RT-qPCR and Western blot techniques to evaluate the osteogenic differentiation of BMSCs. The results demonstrated that the expression levels of osteogenic differentiation markers were increased in the METTL16-KO#1 and METTL16-KO#2 group compared to the METTL16-WT group. Conversely, the osteogenic differentiation marker expression levels decreased in the oe-METTL16 group compared to the oe-NC group (Fig. 2E–G). The Oil Red O staining results and RT-qPCR analysis for each treatment group indicate that METTL16 also plays a regulatory role in adipogenic differentiation (Fig. 2H, I).

The results above show an upregulation of METTL16 in osteoporotic mice. Additionally, it inhibits the osteogenic differentiation of BMSCs.

### Identification of PPARγ as a key downstream target of METTL16 in BMSCs and its association with ferroptosis and m6A methylation pathways

Previous research has indicated a close relationship between iron deficiency and the occurrence and progression of osteoporosis. Additionally, effective regulation of iron levels could help prevent osteoporosis [16, 17]. The ferroptosis gene has been shown to inhibit the process of osteogenic differentiation by inducing ferroptosis in bone marrow mesenchymal stem cells (BMSCs) [14, 15].

**Fig. 3:**
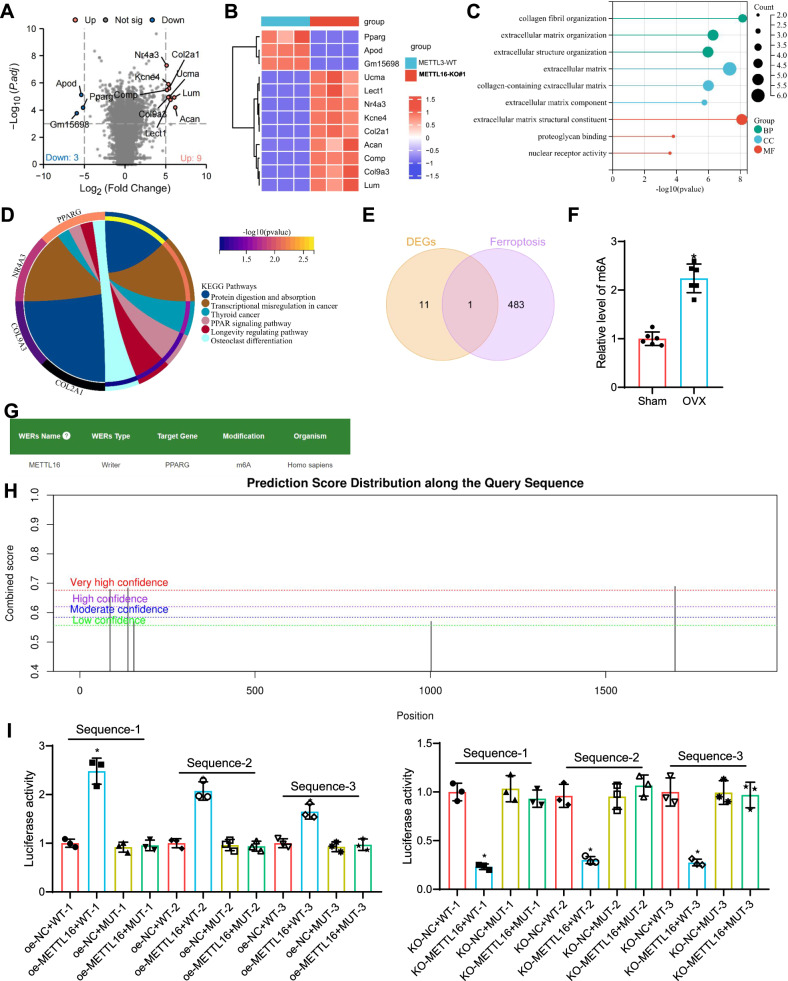
Screening of downstream target genes of METTL16. **A** Volcano plots showing differentially expressed genes between 3 METTL16-WT and 3 METTL16-KO#1 BMSCs in high-throughput sequencing data; **B** Heatmap showing the expression levels of 12 differentially expressed genes in the sequencing data; **C** Bar plot of GO enrichment analysis for the 12 differentially expressed genes, with green, blue, and red representing BP, CC, and MF respectively; **D** KEGG pathway analysis for the 12 differentially expressed genes; **E** Venn diagram showing the intersection between differentially expressed genes in the sequencing data and ferroptosis related genes; **F** Detection of overall m6A levels in bone tissues of the sham and OVX groups; **G** Prediction results of potential m6A-modified target genes of METTL16 by the RM2Target database; **H** Prediction results of potential m6A methylation sites of PPARγ by the SRAMP database. **I** Dual-luciferase reporter gene assays were used to investigate the targeting relationship between METTL16 and different sites on PPARγ in cells. * denotes significance compared to oe-NC + WT-1, oe-NC + WT-2, or oe-NC + WT-3 groups with *p* < 0.05, while # denotes significance compared to the oe-METTL16 + WT-1 group with *p* < 0.05; the cell experiments were repeated three times.

To further identify the downstream target genes of METTL16, we conducted high-throughput sequencing on bone marrow mesenchymal stem cells (BMSCs) from both the METTL16-WT and METTL16-KO groups. Following processing and differential analysis, the findings reveal 12 noteworthy disparities in gene expression (Fig. 3A). The expression patterns of these 12 genes are displayed in Fig. 3B.

Gene enrichment analysis was conducted on the samples, revealing that the differentially expressed genes were primarily enriched in biological processes related to collagen fiber, extracellular matrix, and extracellular structural tissue (Fig. 3C). The results of the KEGG enrichment analysis showed that the differentially expressed genes were primarily enriched in pathways such as protein digestion and absorption, PPAR signaling pathway, and osteoclast differentiation (Fig. 3D). We intersected the genes associated with ferroptosis, obtained from the ferroptosis database FerrDb V2, and identified key genes, including PPARγ (Fig. 3E).

Epigenetic modifications regulate gene expression and translation, thereby influencing cell development and differentiation [68]. Recent research has indicated that m6A methylation plays a role in the pathogenesis of bone-related disorders, such as osteoporosis [69]. And osteoarthritis [70]. Furthermore, m6A RNA methylation plays a pivotal role in regulating bone formation and absorption through its impact on cytokines, hormones, and signaling pathways. METTL16 is a well-established m6A writer that regulates the m6A methylation of downstream target genes, thereby promoting their expression [66, 71, 72]. Therefore, we examined the m6A levels in bone tissues of the control and OVX mice, and the results showed that the m6A modification level was significantly upregulated in the bone tissues of OVX mice (Fig. 3F).

According to the RM2Target database, METTL16 may regulate the m6A modification of PPARγ (Fig. 3G). The prediction results from the SRAMP database reveal the presence of five binding sites for PPARγ, suggesting the potential for m6A methylation in PPARγ under the regulation of m6A writers (Fig. 3H). Three highly confident predicted sites were selected for Me-RIP experiments. Subsequently, precise primers targeting these three m6A modification sites were used for RT-qPCR. Luciferase reporter gene assays revealed that overexpression of METTL16 significantly enhanced the wild-type PPARγ activity at Sequence-1, Sequence-2, and Sequence-3 sites, without affecting the mutant PPARγ activity. Notably, the trend was most significant at the Sequence-1 site, indicating that this site is the primary m6A modification site on PPARγ mRNA regulated by METTL16 (Fig. 3I). In summary, we identified PPARγ, a downstream ferroptosis-related target gene of METTL16, and confirmed the METTL16-mediated m6A regulatory site on PPARγ mRNA.

The results above have led us to identify a target gene downstream of METTL16 associated with iron-related mortality.

### METTL16 modulates m6A methylation of PPARγ, influencing its expression and post-transcriptional stability in BMSCs

**Fig. 4:**
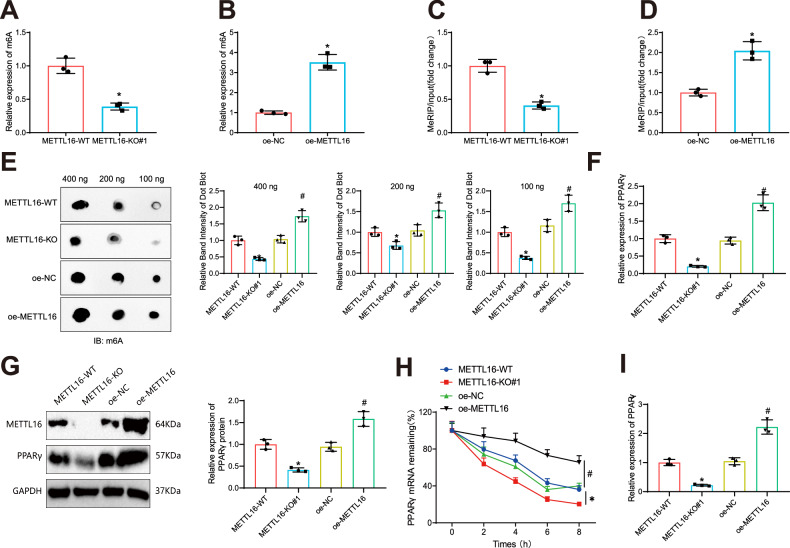
Effect of METTL16 on m6A modification of PPARγ in BMSCs. **A**, **B** Detection results of total m6A levels in BMSCs of each group; **C**, **D** MeRIP-qPCR analysis of the influence of METTL16 knockdown or overexpression on m6A levels of PPARγ in BMSCs; **E** Dot blot experiment to study the effect of METTL16 knockdown or overexpression on the total m6A levels, and quantitative analysis; **F** Gene expression levels of PPARγ in each group of BMSCs detected by RT-qPCR; **G** Protein expression levels of PPARγ in each group of BMSCs detected by Western blot; **H** Stability of PPARγ in each group of BMSCs tested by Actinomycin D experiment; **I** PAR-CLIP experiment on the binding of METTL16 and PPARγ. * indicates *p* < 0.05 compared to the METTL16-WT group, # indicates *p* < 0.05 compared to the oe-NC group, cell experiments were repeated three times.

To further validate the regulatory function of METTL16 in m6A methylation of PPARγ, we initially investigated the impact of METTL16 knockout or overexpression on the overall m6A content in BMSCs. The results demonstrate that the knockout of METTL16 led to a substantial reduction in total m6A levels, whereas overexpression of METTL16 resulted in a remarkable increase in total m6A levels (Fig. 4A, B).

MeRIP-qPCR experiments revealed that the knockout or overexpression of METTL16 in BMSCs resulted in a substantial decrease or increase in the m6A levels of PPARγ mRNA (Fig. 4C–D). The results of the Dot blot experiment demonstrated that knocking out METTL16 led to a decrease in m6A methylation levels in BMSCs while overexpressing METTL16 resulted in an increase in m6A methylation levels in transfected BMSCs (Fig. 4E).

The results from RT-qPCR and Western blot analyses demonstrated that knockout of METTL16 led to a reduction in both mRNA and protein expression levels of PPARγ. Conversely, overexpression of METTL16 resulted in a substantial increase in the mRNA and protein expression levels of PPARγ, as illustrated in Fig. 4F, G. Overexpression of METTL16 extended the half-life of PPARγ mRNA (Fig. 4H). Using PAR-CLIP experiments, we examined the binding of METTL16 and PPARγ in BMSCs, with METTL16 as the antibody. The results of PPARγ expression levels pulled down by the METTL16 antibody showed a significant increase in the oe-METTL16 group compared to the oe-NC group, and a significant decrease in the METTL16-KO group compared to the METTL16-WT group (Fig. 4I). These findings indicate that METTL16 can regulate the m6A modification of PPARγ and promote its post-transcriptional modification.

These findings suggest that METTL16 regulates the m6A modification of PPARγ and promotes its post-transcriptional modification.

### PPARγ‘s key role in modulating ferroptosis: impact on BMSCs viability, oxidative balance, and mitochondrial integrity

To investigate the regulatory role of PPARγ in the ferroptosis of BMSCs, we designed three shRNA sequences specific to target PPARγ. The knockdown effect of sh-PPARγ was confirmed, and for subsequent experiments, sh-PPARγ-3, with the highest knockdown efficiency, was chosen (Fig. S6A). PPARγ was depleted in BMSCs cells, and subsequently, we conducted CCK-8, scratch assay, Transwell assay, and flow cytometry. The findings revealed that compared to the sh-NC group, the sh-PPARγ group exhibited enhanced BMSCs cell viability, migration, and invasion abilities while the apoptosis rate decreased (Fig. S6B-E).

We identified specific indicators associated with ferroptosis, including lipid ROS, MDA, GSH, GSSG, and Fe^2+^ [73, 74]. Initially, we employed the FerroOrange probe to examine the expression of iron metabolism-related genes to investigate the regulatory function of PPARγ in iron homeostasis, as depicted in Fig. S7A-B. Subsequently, a reduction in intracellular Fe^2+^ was observed within a 24-hour after the knockdown of sh-PPARγ.

To investigate the influence of PPARγ on redox balance, we compared markers of oxidative stress and peroxidation products. The sh-PPARγ group increased the GSH/GSSG ratio, indicating reduced oxidative stress levels (Fig. S7C). Both confocal laser scanning microscopy examination and flow cytometry fluorescence quantification results reveal that BMSCs treated with PPARγ knockdown exhibit reduced production of ROS (Fig. S7D-E). ROS present in the cytoplasm can oxidize polyunsaturated lipids in the cellular membrane, resulting in the production of final products such as MDA [61].

The results demonstrated a decrease in the levels of MDA in the sh-PPARγ group (Fig. S7F). The level of lipid peroxidation, as assessed by C11-BODIPY581/591, was decreased following the knockdown of sh-PPARγ (Fig. S7G). Furthermore, we investigated the expression of GPX4 and SLC7A11, which serve as markers of ferroptosis and indicate the ability for oxidative repair [75, 76]. The mRNA transcription levels of sh-PPARγ on GPX4 and SLC7A11 showed a significant increase (Fig. S7H-I). Additionally, there was a notable elevation in the protein levels (Fig. S7J).

Iron toxicity is associated with distinct alterations in mitochondrial morphology [77, 78]. Fig. S7K demonstrates the observation of mitochondrial damage repair in BMSCs of the sh-PPARγ group. Conversely, the BMSCs of the sh-NC group exhibited mitochondrial atrophy, ridge reduction, and an increase in membrane density. Detection of JC-1 showed that the downregulation of PPARγ decreased the mitochondrial membrane potential of BMSCs, as illustrated in Fig. S7L. This study suggests that PPARγ may regulate ferroptosis in BMSCs by influencing mitochondrial function.

To summarize, these findings further confirm the role of PPARγ in regulating and inducing ferroptosis in BMSCs.

### METTL16/PPARγ axis impedes osteogenic differentiation of BMSCs through promotion of ferroptosis: experimental validation and molecular insights

To examine the influence of the METTL16/PPARγ axis on the osteogenic differentiation of BMSCs through cell death caused by iron, we conducted knockout experiments targeting METTL16 and/or overexpressed PPARγ in BMSCs. Using RT-qPCR, the study examined the expression of METTL16 and PPARγ. The results demonstrated a decrease in the expression of both METTL16 and PPARγ in the METTL16-KO + oe-NC group compared to the METTL16-WT + oe-NC group. Furthermore, no change was observed in the expression level of METTL16 in the METTL16-KO + oe-PPARγ group compared to the METTL16-KO + oe-NC group, while there was an increase in the expression of PPARγ (Fig. S8A-B).

The results obtained from the CCK-8, scratch assay, Transwell assay, and flow cytometry demonstrated that the cell viability, migration, and invasion capabilities of BMSCs were increased in the METTL16-KO + oe-NC group compared to the METTL16-WT + oe-NC group. Moreover, the apoptosis rate was decreased. On the other hand, in the METTL16-KO + oe-PPARγ group, the cell viability, migration, and invasion capabilities of BMSCs were reduced, while apoptosis was increased when compared to the METTL16-KO + oe-NC group (Fig. S8C-F).

**Fig. 5:**
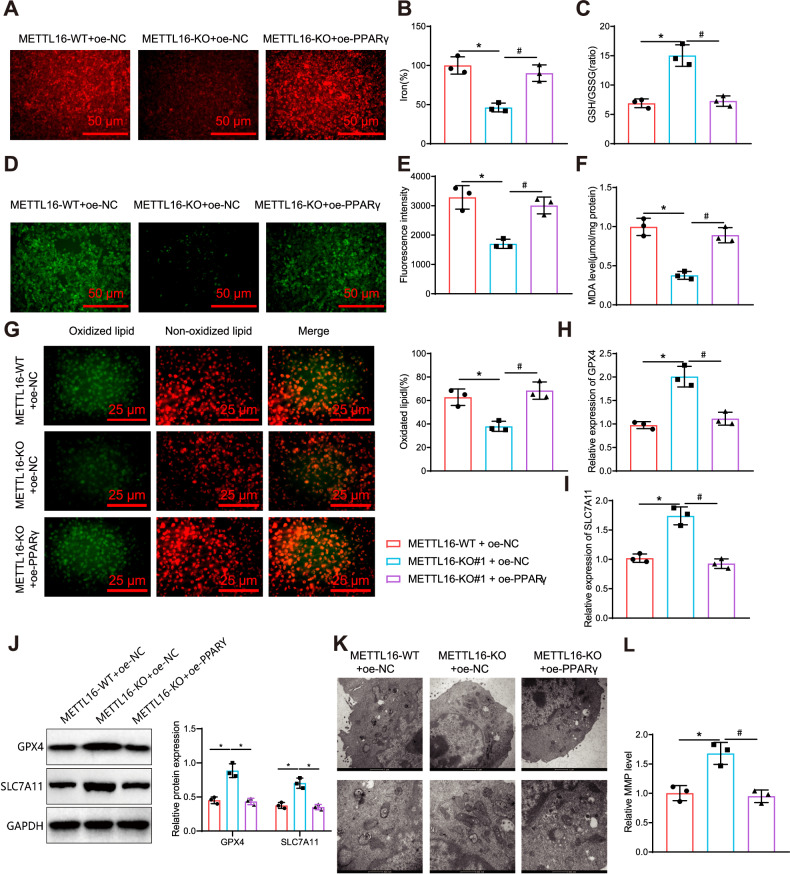
Effect of the METTL16/PPARγ axis on ferroptosis in BMSCs. **A** Representative fluorescence images of intracellular iron levels in BMSCs transfected with different lentiviruses after 12 h using FerroOrange staining (Scale bar=50 μm); **B** Statistical results of iron content in BMSCs of each group using a test kit; **C** GSH/GSSG ratio in BMSCs after different treatments; **D**, **E** Visualization of ROS production in BMSCs using DCFH-DA and statistical analysis by flow cytometry, Scale bar = 50 μm; **F** Detection of MDA content in each group; **G** Representative confocal images of BMSCs stained with C11-BODIPY 581/591. Red indicates non-oxidized lipids, green represents oxidized lipids (Scale bar = 25 μm); **H**, **I** Transcriptional levels of ferroptosis-related genes SLC7A11 and GPX4 detected by RT-qPCR; **J** Western blot to detect the protein expression levels of ferroptosis-related genes SLC7A11 and GPX4 in each group of BMSCs; **K** Observation of mitochondrial morphology in cells by transmission electron microscopy, Scale bar = 1 μm; **L** Detection of mitochondrial membrane potential (MMP) in each group of cells using JC-1. * indicates *p* < 0.05 compared to the METTL16-WT + oe-NC group, # indicates *p* < 0.05 compared to the METTL16-KO + oe-NC group, cell experiments were repeated three times.

Further examination of ferroptosis-specific indicators revealed that the METTL16-KO + oe-NC group exhibited a reduction in intracellular Fe^2+^, ROS, MDA, and lipid peroxidation levels when compared to the METTL16-WT + oe-NC group. The METTL16-KO + oe-NC group also demonstrated a notable increase in the GSH/GSSG ratio. Conversely, the METTL16-KO + oe-PPARγ group exhibited an increase in intracellular Fe^2+^, ROS, and MDA levels, as well as lipid peroxidation levels, accompanied by a substantial decrease in the GSH/GSSG ratio, when compared to the METTL16-KO + oe-NC group (Fig. 5A–G).

We analyzed the expression of GPX4 and SLC7A11 to further validate our findings. The results from RT-qPCR and Western blot demonstrate that the METTL16-KO + oe-NC group had higher mRNA and protein expression levels of both GPX4 and SLC7A11 compared to the METTL16-WT + oe-NC group. On the other hand, the METTL16-KO + oe-PPARγ group showed lower mRNA and protein expression levels of GPX4 and SLC7A11 compared to the METTL16-KO + oe-NC group (Fig. 5H–J).

Furthermore, as depicted in Fig. 5K, the METTL16-KO + oe-NC group demonstrated repaired mitochondrial damage, increased cristae, and reduced membrane density compared to the METTL16-WT + oe-NC group. Conversely, the METTL16-KO + oe-PPARγ group exhibited mitochondrial atrophy, decreased cristae, and elevated membrane density compared to the METTL16-KO + oe-NC group. The results from the JC-1 detection indicated that, in comparison to the METTL16-WT + oe-NC group, the mitochondrial membrane potential of BMSCs in the METTL16-KO + oe-NC group decreased. Furthermore, in contrast to the METTL16-KO + oe-NC group, the mitochondrial membrane potential of BMSCs increased in the METTL16-KO + oe-PPARγ group (Fig. 5L).

**Fig. 6:**
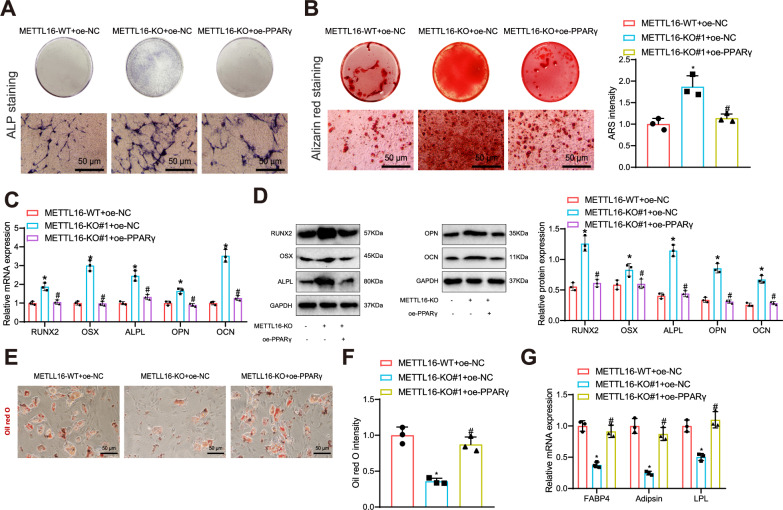
Impact of the METTL16/PPARγ Axis on BMSCs Osteogenic Differentiation. **A** ALP staining to measure the ALP activity in different groups of BMSCs, scale bar: 50 μm. **B** ARS staining to assess osteogenic differentiation in different groups of BMSCs, scale bar: 50 μm. **C** RT-qPCR analysis of the gene expression levels of osteogenic markers (RUNX2, Osterix, ALPL, OPN, OCN) in different groups of BMSCs. **D** Western blot analysis of the protein expression levels of osteogenic markers (RUNX2, Osterix, ALPL, OPN, OCN) in different groups of BMSCs. **E** Oil Red O staining to analyze adipogenic differentiation in bone tissues of each group, scale bar: 50 μm. **F** Quantitative analysis of adipogenic differentiation in bone tissues of each group. **G** RT-qPCR analysis of gene expression levels of adipogenic differentiation markers FABP4, Adipsin, and LPL in BMSCs of each group. * indicates difference compared to METTL16-WT + oe-NC group (*p* < 0.05). # indicates difference compared to METTL16-KO + oe-NC group (*p* < 0.05). Cell experiments were repeated 3 times.

The results of ALP staining and ARS staining revealed that the METTL16-KO + oe-NC group exhibited enhanced ALP and ARS staining, which promoted the osteogenic differentiation of *BMSCs* (BMSCs), in comparison to the METTL16-WT + oe-NC group. Furthermore, the osteogenic differentiation level of BMSCs in the METTL16-KO + oe-PPARγ group was lower than that of the METTL16-KO + oe-NC group (Fig. 6A, B).

The expression levels of osteogenic differentiation markers in BMSCs were assessed using RT-qPCR and Western blot analysis. The results demonstrated that the METTL16-KO + oe-NC group exhibited increased expression levels of osteogenic differentiation markers compared to the METTL16-WT + oe-NC group. Conversely, the METTL16-KO + oe-PPARγ group displayed decreased expression levels of osteogenic differentiation markers relative to the METTL16-KO + oe-NC group (Fig. 6C, D). The Oil Red O staining results showed a significant decrease in staining signal after METTL16 knockout, indicating that METTL16 knockdown inhibited adipogenic differentiation of BMSCs. In contrast, the level of adipogenic differentiation of BMSCs in the METTL16-KO + oe-PPARγ group was significantly restored compared to the METTL16-KO + oe-NC group (Fig. 6E, F). RT-qPCR analysis of the expression levels of adipogenic differentiation markers in BMSCs showed a significant decrease in the METTL16-KO + oe-NC group compared to the METTL16-WT + oe-NC group, whereas a significant increase in adipogenic marker expression levels was observed in the METTL16-KO + oe-PPARγ group compared to the METTL16-KO + oe-NC group (Fig. 6G).

The above findings suggest that the METTL16/PPARγ axis hinders the osteogenic differentiation of BMSCs by promoting ferroptosis.

### METTL16/PPARγ axis exacerbates osteoporosis in mice via ferroptosis: molecular insights and bone morphological evidence

**Fig. 7:**
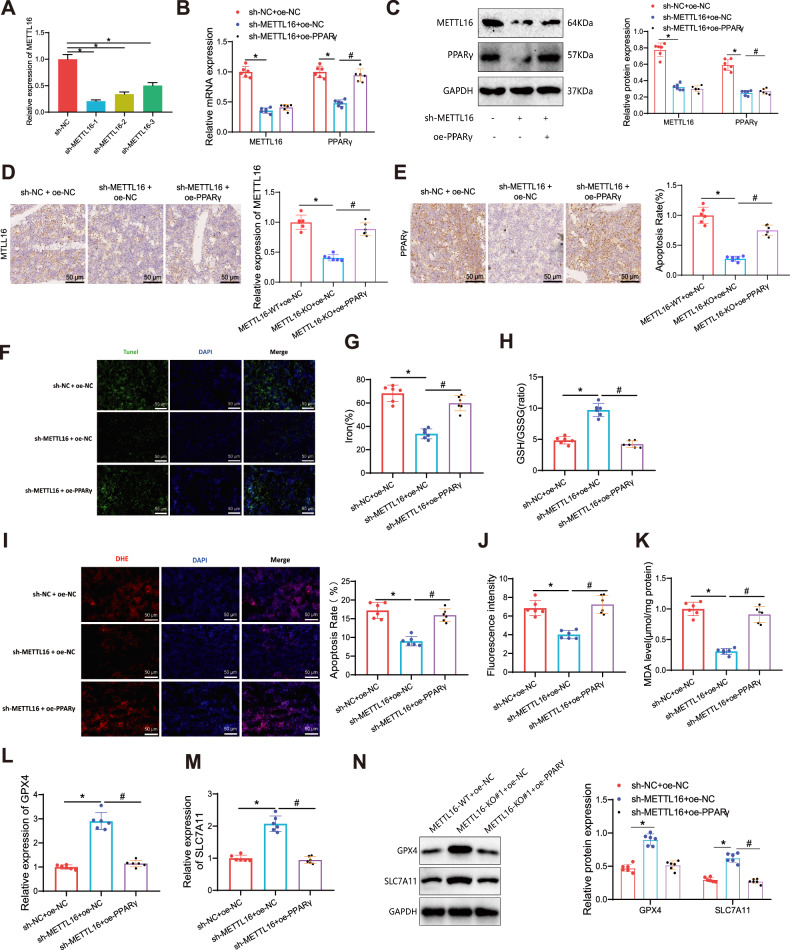
Impact of the METTL16/PPARγ axis on mouse bone tissue cells ferroptosis. **A** RT-qPCR analysis of the silencing efficiency of three METTL16 shRNAs; **B**, **C** RT-qPCR and Western blot analysis of METTL16 and PPARγ protein expression levels in the bone tissues of each group of mice; **D**, **E** IHC detection of METTL16 and PPARγ expression (Scale bar = 50 μm); **F** TUNEL analysis of cell apoptosis rate in mouse bone tissues of each group (Scale bar = 50 μm); **G** Statistical results of iron content in mouse bone tissues of each group, measured using assay kits; **H** GSH/GSSG ratio in mouse bone tissues after different treatments; **I**, **J** Visualization and statistical analysis of ROS production in mouse bone tissues using fluorescent probes DHE (red) and DAPI (blue), Scale bar = 50 μm; **K** MDA content in mouse bone tissues of each group; **L**, **M** qRT-PCR analysis of transcription levels of ferroptosis-related genes SLC7A11 and GPX4; **N** Western blot analysis of protein expression levels of ferroptosis-related genes SLC7A11 and GPX4 in each group of BMSCs. * indicates difference compared to sh-NC + oe-NC group or sh-NC group (*p* < 0.05). # indicates difference compared to sh-METTL16 + oe-NC group (*p* < 0.05). Cell experiments were repeated 3 times, with 6 mice in each group.

The in vitro cell experiment above confirmed that the METTL16/PPARγ axis suppresses the osteogenic differentiation of BMSCs by inducing ferroptosis. To investigate the impact of this mechanism on osteoporosis in mice, an osteoporotic mouse model was constructed using the OVX method. Lentiviruses were intravenously injected to simultaneously knock down METTL16 and/or overexpress PPARγ. Three shRNA sequences targeting METTL16 were designed, followed by validation of the knockdown effect using in vitro cell models. Choose the most efficient sh-METTL16-1 sequence, from now on referred to as sh-METTL16, for the following experiments (Fig. 7A).

Firstly, RT-qPCR and Western blot analysis revealed a decrease in both the mRNA and protein expression levels of METTL16 and PPARγ in the sh-METTL16 + oe-NC group of mouse bone tissues, compared to the sh-NC + oe-NC group. Compared to the sh-METTL16 + oe-NC group, METTL16 mRNA and protein expression in bone tissue showed no alterations in sh-METTL16 + oe-PPARγ mice. However, there was an increase in the expression levels of PPARγ mRNA and protein (Fig. 7B, C). First, the RT-qPCR and Western blot results showed that the mRNA and protein expression levels of METTL16 and PPARγ were significantly reduced in the bone tissues of the sh-METTL16 + oe-NC group compared to the sh-NC + oe-NC group. Compared to the sh-METTL16 + oe-NC group, there was no significant change in METTL16 mRNA and protein expression in the sh-METTL16 + oe-PPARγ group, while the mRNA and protein expression levels of PPARγ were significantly elevated (Fig. 7B, C). Immunohistochemical (IHC) staining of bone tissue samples showed that staining signals for METTL16 and PPARγ were significantly decreased in the bone tissues of the sh-METTL16 + oe-NC group compared to the sh-NC + oe-NC group. Compared to the sh-METTL16 + oe-NC group, the METTL16 staining signal in the bone tissues of the sh-METTL16 + oe-PPARγ group showed no significant change, whereas the PPARγ staining signal was significantly increased (Fig. 7D, E). TUNEL staining of bone tissue in mice showed a reduced apoptosis rate in the sh-METTL16 + oe-NC group. Compared to the sh-METTL16 + oe-NC group, the sh-METTL16 + oe-PPARγ group displayed an increased apoptosis rate (Fig. 7F).

Additionally, we collected bone tissue from mice to measure ROS, MDA, the GSH/GSSG ratio, and iron content. It was done to assess the degree of cell death caused by iron. The results indicated that in the bone tissue of mice in the sh-METTL16 + oe-NC group, there was a reduction in the levels of Fe^2+^, ROS, and MDA, while the ratio of GSH/GSSG increased compared to the sh-NC + oe-NC group. Compared to the sh-METTL16 + oe-NC group, the levels of Fe^2+^, ROS, and MDA increased in the bone tissue of mice in the sh-METTL16 + oe-PPARγ group. Conversely, the GSH/GSSG ratio decreased (Fig. 7G–K).

Furthermore, we observed the expression of GPX4 and SLC7A11 factors implicated in ferroptosis. The RT-qPCR results showed an elevation in the mRNA expression levels of GPX4 and SLC7A11 in mouse bone tissue in the sh-METTL16 + oe-NC group. Compared to the sh-NC + oe-NC group, the mRNA expression levels of GPX4 and SLC7A11 in the mouse bone tissue of the sh-METTL16 + oe-PPARγ group showed a decrease (Fig. 7L, M). Protein expression levels of ferroptosis-related genes SLC7A11 and GPX4 in BMSCs were assessed in this study. Compared to the sh-NC+oe-NC group, a significant elevation was observed in both GPX4 and SLC7A11 in the sh-METTL16+oe-NC group. Conversely, there were no significant changes in GPX4 and SLC7A11 protein levels in the sh-METTL16+oe-PPARγ group compared to the sh-NC+oe-NC group. These results suggest that overexpression of PPARγ may mitigate the increase in GPX4 and SLC7A11 protein levels caused by METTL16 knockdown (Fig. 7N).

**Fig. 8:**
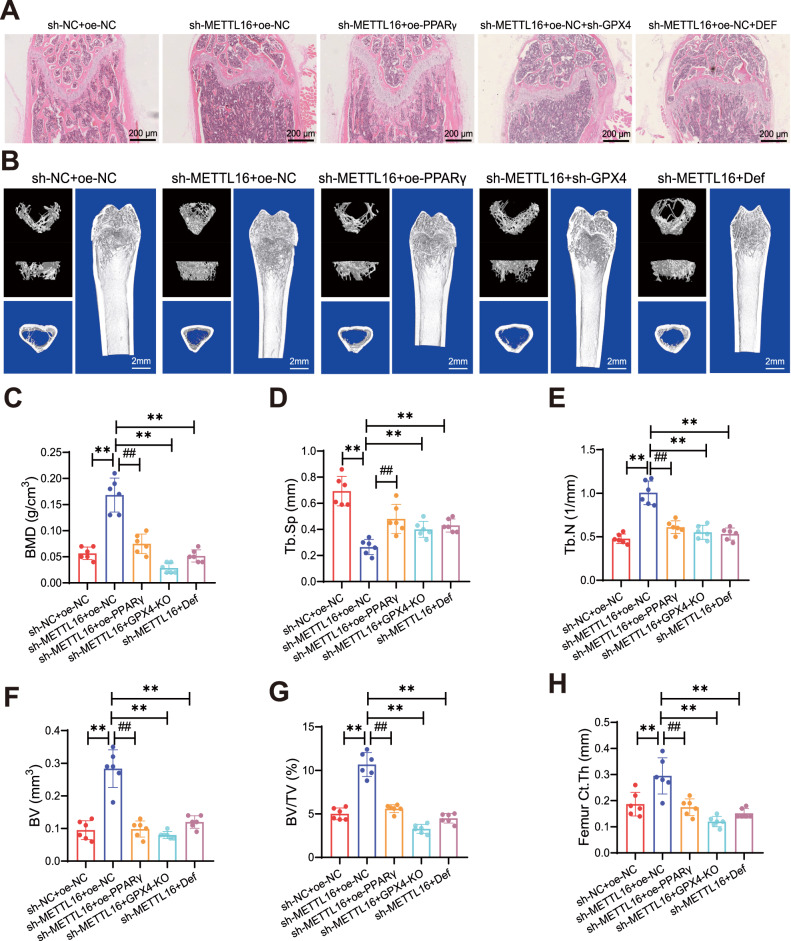
Impact of the METTL16/PPARγ Axis on Osteoporotic Mouse Model. **A** H&E staining of femoral mid-shaft in different groups of mice, scale bar: 200 μm. **B** Micro-CT images of different groups of mice. **C**–**H** Micro-CT analysis and statistics of BMD, Tb.Sp, Tb.N, BV, BV/TV, and ct.Th in different groups of mice. Each group consisted of 6 mice. * and # indicate comparisons between two groups. *p < 0.05, **p < 0.01, #*p* < 0.05, ##*p* < 0.01.

H&E staining was used to observe the distal femur of mice. The results showed that in the sh-METTL16 + oe-NC group, the trabecular bone in the distal femur was thicker, more regularly arranged, denser, and had a clear and intact structure compared to the sh-NC + oe-NC group, whereas the distal femur of the sh-METTL16 + oe-NC group showed the opposite trend. Rescue experiments using the ferroptosis inhibitor Deferasirox or GPX4 knockdown significantly inhibited the impact of the METTL16/PPARγ axis on bone tissue morphology (Fig. 8A).

Micro-CT was used to observe BMD, BV/TV, Tb.N, BV, Tb.Sp, and ct.Th at the distal right femur in mice. In the sh-METTL16 + oe-NC group, the trabecular bone was thicker, the trabecular structure was more orderly, connectivity was increased, separation was reduced, cortical bone became thicker, and BMD, BV/TV, Tb.N, BV, and ct.Th were all significantly increased, while Tb.Sp was significantly decreased. In contrast, the sh-METTL16 + oe-PPARγ group exhibited thinner trabecular bone, disordered trabecular structure, decreased connectivity, increased separation, thinner cortical bone, significantly reduced BMD, BV/TV, Tb.N, BV, and ct.Th, and significantly increased Tb.Sp. Treatment with Deferasirox or GPX4 knockdown restored these phenotypes (Fig. 8B–H).

The results above demonstrate that the METTL16/PPARγ axis promotes osteoporosis in mice through ferroptosis induction.

## Discussion

This study found that METTL16-mediated PPARγ m6A methylation plays a crucial role in both ferroptosis and osteogenic differentiation of BMSCs [66]. This finding is consistent with prior research, highlighting the significance of m6A methylation in cellular function and regulation of gene expression [19]. However, in contrast to prior studies, we have discovered a direct correlation between METTL16 and PPARγ and extensively investigated its precise involvement in the pathogenesis of osteoporosis.

Ferroptosis is a novel form of cell death that has garnered attention in prior studies, specifically due to its association with ROS and mitochondrial function [79–82]. In our study, we have elucidated the occurrence of iron-death in BMSCs and extensively investigated the regulatory role of the METTL16/PPARγ axis on this process, leading to the inhibition of BMSCs osteogenic differentiation. This offers a novel perspective for understanding the molecular mechanisms of osteoporosis.

Regarding PPARγ, it is a ligand-activated transcription factor involved in lipid metabolism, promoting lipid synthesis for energy storage [83]. Currently, there is limited research on the regulatory role of PPARγ in iron-death. A study has shown that PPARγ can promote iron-death in dendritic cells, impairing anti-tumor immunity in mice [84], consistent with our findings that PPARγ can mediate iron-death. Another study found that another subtype of PPAR, PPARα, mediated cancer cell iron-death through lipid reshaping [85], suggesting that lipid metabolism may also be one of the pathways through which PPARγ promotes BMSCs iron-death. These studies align with our conclusion that PPARγ promotes iron-death. Based on existing research, we hypothesize that in the context of osteoporosis, the regulatory mechanism of PPARγ on iron-death may involve PPARγ as a transcription factor regulating the expression of lipid metabolism genes, leading to lipid reshaping in BMSCs, which triggers iron-death. Subsequently, we will conduct a new study to explore the impact of PPARγ on lipid metabolism in the context of osteoporosis to elucidate this hypothesis.

Moreover, lipid metabolism abnormalities have been shown to promote osteoporosis. Lipid metabolism abnormalities influence bone microenvironment homeostasis through interorgan communication, promoting the differentiation of mesenchymal stem cells into adipocytes and inhibiting their directed differentiation into osteoblasts. Additionally, lipid metabolism disorders can stimulate osteoblasts to secrete factors like nuclear factor κB ligand receptor activators, stimulating osteoclast differentiation and affecting bone metabolism balance [86]. A study on bone marrow mesenchymal stem cells indicated that upregulation of PPAR-γ, by binding with lysine 4 of tri-methylated histone H3 (h3k4me3), activated the transcription of downstream lipid genes, disrupting the balance between osteogenic and adipogenic differentiation, thereby affecting bone health [87]. Therefore, METTL16 may also mediate lipid metabolism abnormalities through PPARγ, a lipid metabolism regulatory factor, leading to osteoporosis, although further experimental confirmation is needed.

PPARγ is an important nuclear hormone receptor primarily responsible for regulating the formation and maturation of adipocytes [88]. In BMSCs, PPARγ expression is a key signal driving BMSCs toward adipocyte differentiation. When BMSCs undergo appropriate induction stimuli, such as insulin, glucocorticoids, or specific differentiation-inducing factors, PPARγ is activated and collaborates with other transcription factors, such as C/EBPα, to promote the expression of adipose-related genes [89]. PPARγ not only plays a crucial role in adipogenic differentiation of BMSCs but also holds broader significance in metabolic regulation. Research on the role of METTL16 in adipogenic differentiation of BMSCs, however, is relatively limited. Our study confirms that METTL16 influences adipogenic differentiation of BMSCs by regulating PPARγ expression.

On a technical level, this study utilized scRNA-seq to analyze the bone tissue of an osteoporotic mouse model. Unlike traditional bulk RNA-seq, this technique precisely revealed the specific expression of METTL16, an m6A regulatory factor, in bone marrow mesenchymal stem cells, highlighting the critical role of METTL16 in these cells [20]. Additionally, we found that the m6A levels in bone tissue of osteoporotic mice constructed using OVX were significantly upregulated, indicating the essential role of RNA m6A modification in the regulation and progression of osteoporosis.

At the animal model level, by utilizing an osteoporosis mouse model constructed through OVX, we were able to verify the results of our in vitro experiments in an in vivo environment, ensuring the reliability and practical value of our study. This model also provides convenience for future drug screening and intervention strategies research.

**Fig. 9:**
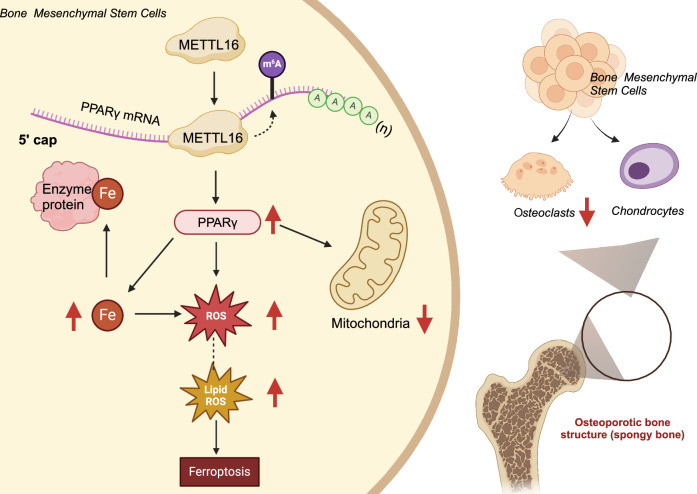
Schematic representation of the molecular mechanism in which METTL16 upregulates PPARγ m6A methylation, leading to ferroptosis of BMSCs and inhibiting their osteogenic differentiation, ultimately causing osteoporosis.

Based on the results above, several preliminary conclusions could be drawn. Specifically, it was found that METTL16 is capable of regulating the m6A modification of the ferroptosis-related gene PPARγ. This regulation facilitates the post-transcriptional modification of PPARγ and its subsequent activation of BMSCs ferroptosis, inhibiting BMSCs’ osteogenic differentiation. Consequently, the occurrence of osteoporosis is further promoted, as illustrated in Fig. 9. Methylation of m6A, an important RNA modification, has been extensively validated in prior research for its role in regulating gene expression and function [18, 19]. This study further deepened our understanding of the relationship between METTL16-mediated m6A methylation of PPARγ and ferroptosis and osteogenic differentiation in BMSCs. Despite the significant findings mentioned above, our research still has some limitations. Firstly, although the impact of the METTL16/PPARγ axis on osteoporosis was observed in C57BL/6 mice, there are physiological differences between mice and humans, so further validation is needed to determine if these results can be directly applied to humans. Additionally, our sample size for scRNA-seq data is limited, posing a potential bias risk that merely provides clues for subsequent studies. Finally, while this study revealed the relationship between METTL16-mediated PPARγ m6A methylation and ferroptosis in BMSCs, a deeper exploration of the molecular mechanisms underlying how PPARγ regulates ferroptosis is required. Citing relevant literature [84, 85, 90] along with the findings of our study, we hypothesize that in the context of osteoporosis, the regulatory mechanism of PPARγ on ferroptosis may involve PPARγ acting as a transcription factor that modulates the expression of lipid metabolism genes, leading to lipid reshaping in BMSCs, thereby triggering ferroptosis. We will conduct a new study to explore the impact of PPARγ on lipid metabolism in the context of osteoporosis to elucidate the aforementioned hypothesis.

Future investigations could further explore the role of the METTL16/PPARγ axis in other diseases, including skeletal disorders or diseases associated with the bone marrow microenvironment, based on the findings of this study. Further exploration through preliminary clinical trials is needed to validate the efficacy of potential intervention strategies targeting the METTL16/PPARγ axis in treating human osteoporosis. The strategy of integrating single-cell technology with Bulk RNA-seq could be further optimized and improved in future research, thereby offering enhanced tools and methods for biomedical research.

## Supplementary information

Original WB images

Supplementary Tables and figures

## Data Availability

Data is available on request from the authors. The data that support the findings of this study are available from the corresponding author upon reasonable request.
