# Supplementary material for: Role of METTL16 in PPARγ methylation and osteogenic differentiation
Source: Cell Death Dis. 2025 Apr 10;16(1):271. doi: 10.1038/s41419-025-07527-x (PMC11986173; doi:10.1038/s41419-025-07527-x)
Supplement: Supplementary file 1 — Original WB images [file 41419_2025_7527_MOESM1_ESM.docx]

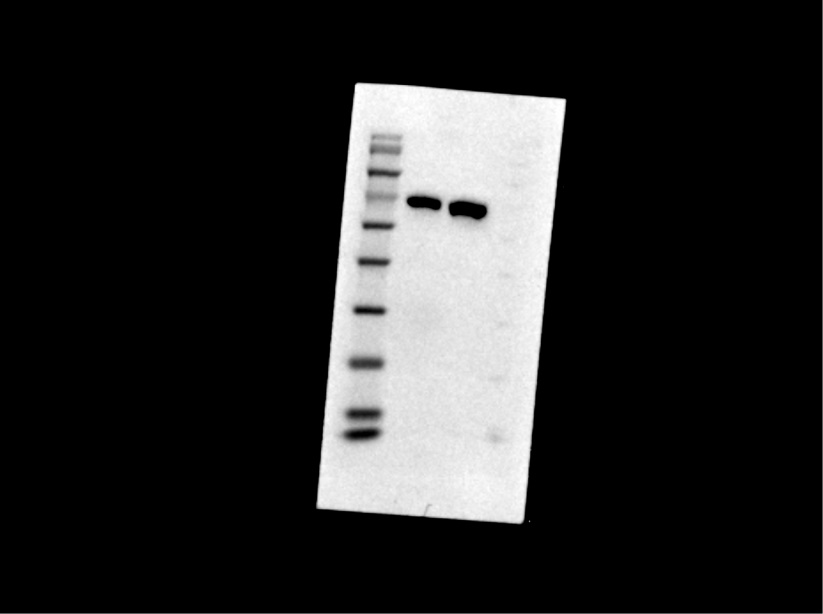


Figure 2B-1


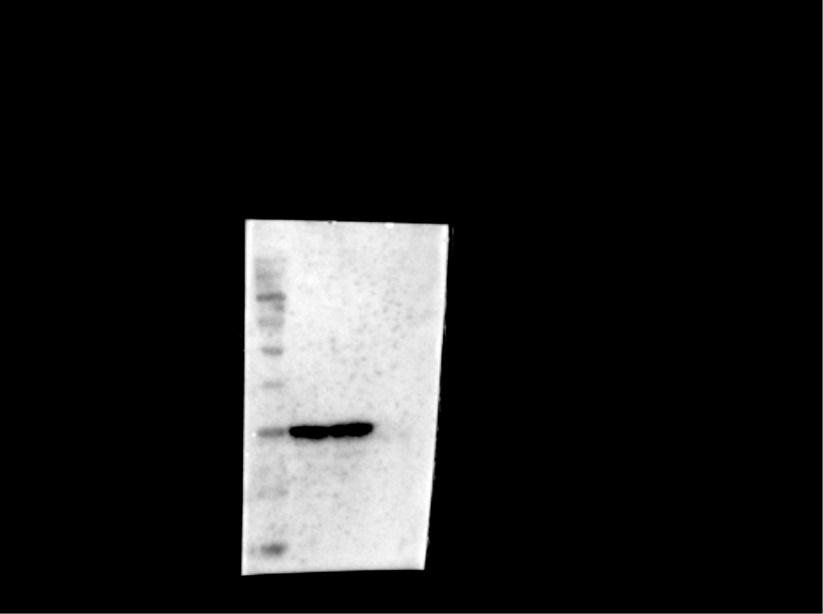


Figure 2B-2


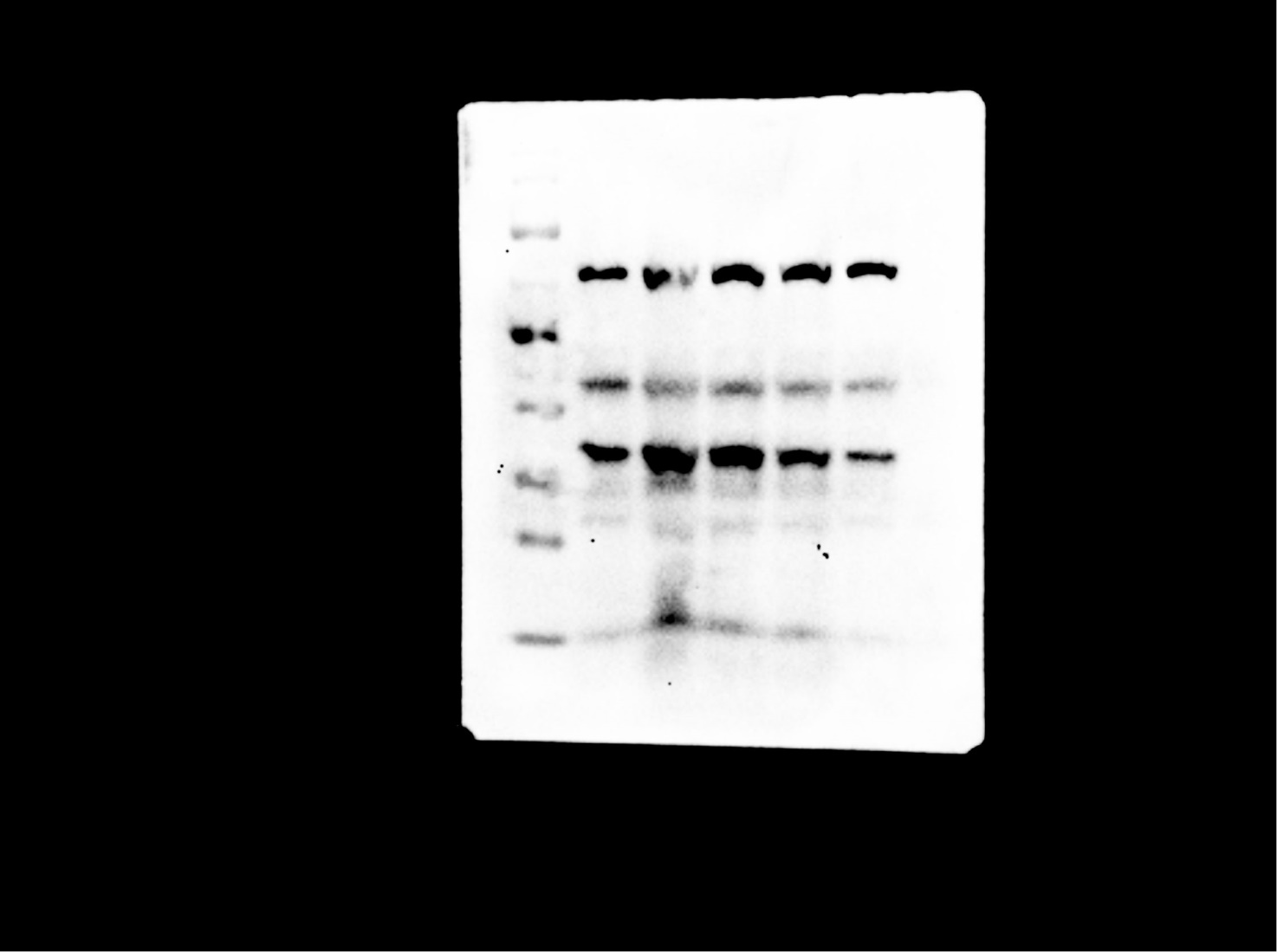


Figure 2G-1


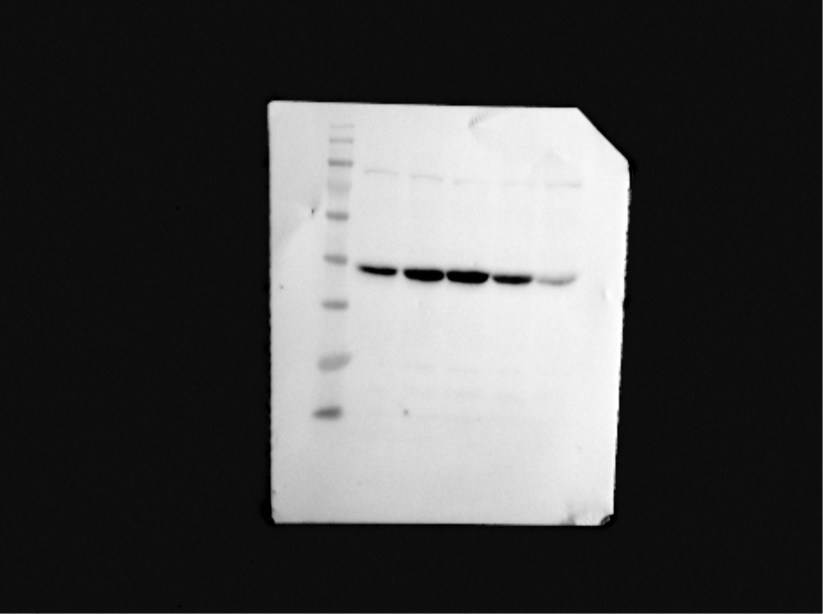


Figure 2G-2


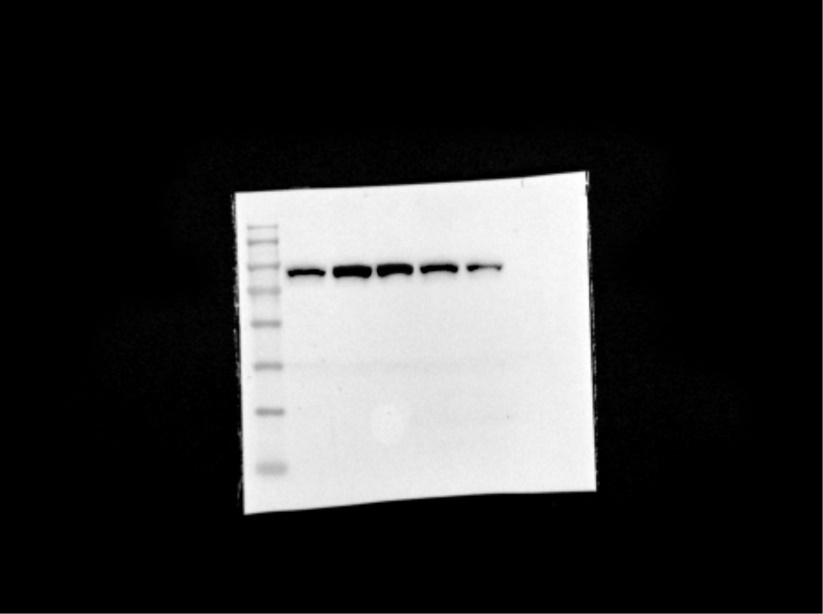


Figure 2G-3


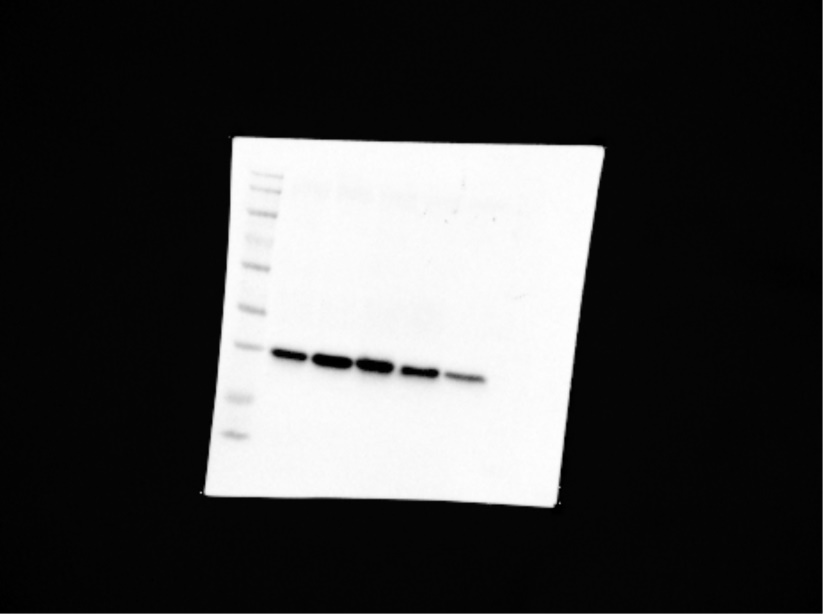


Figure 2G-4


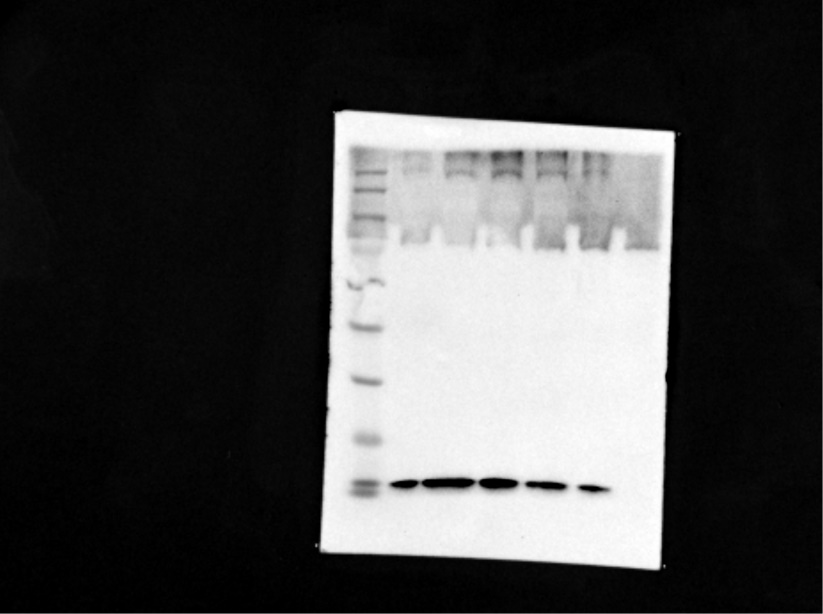


Figure 2G-5


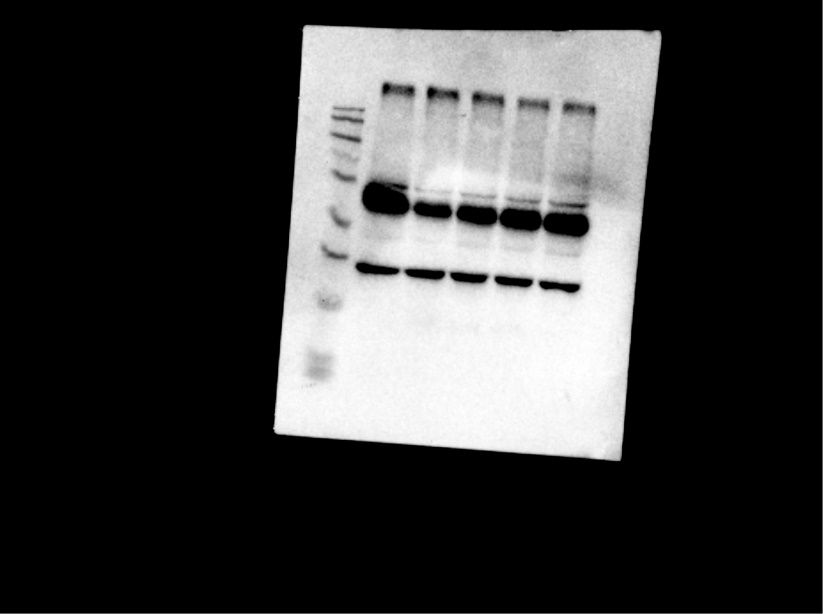


Figure 2G-6


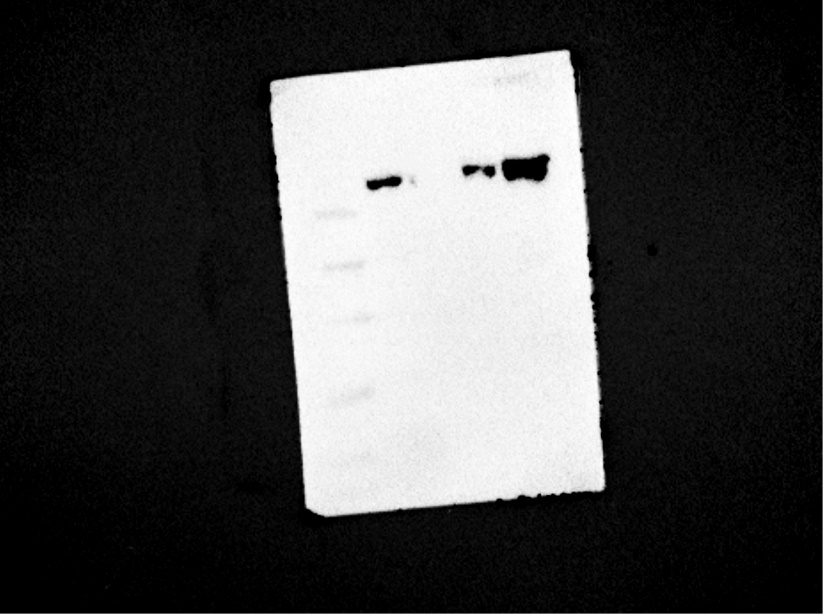


Figure 4G-1


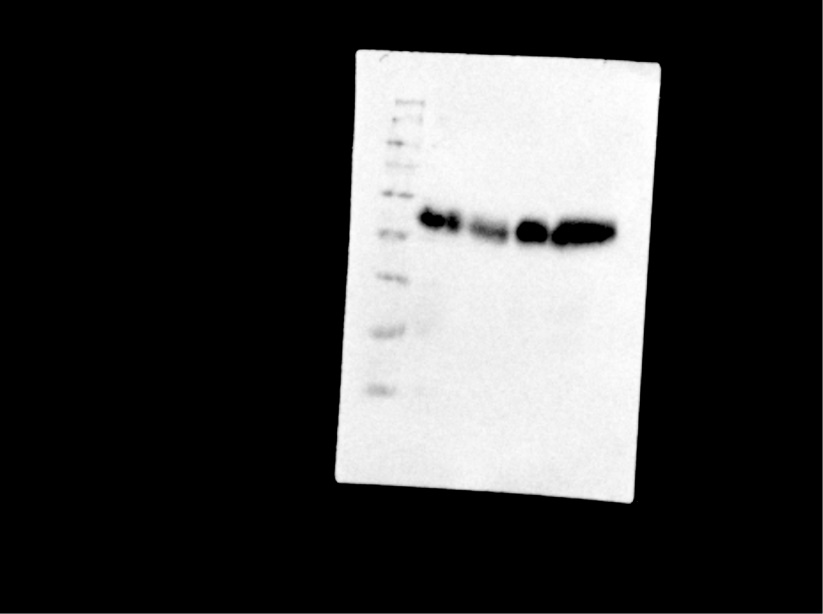


Figure 4G-2


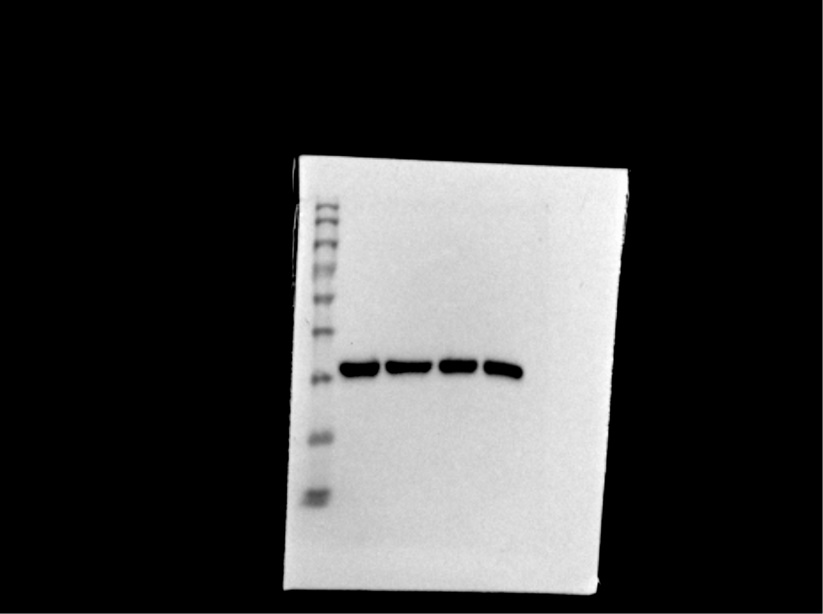


Figure 4G-3


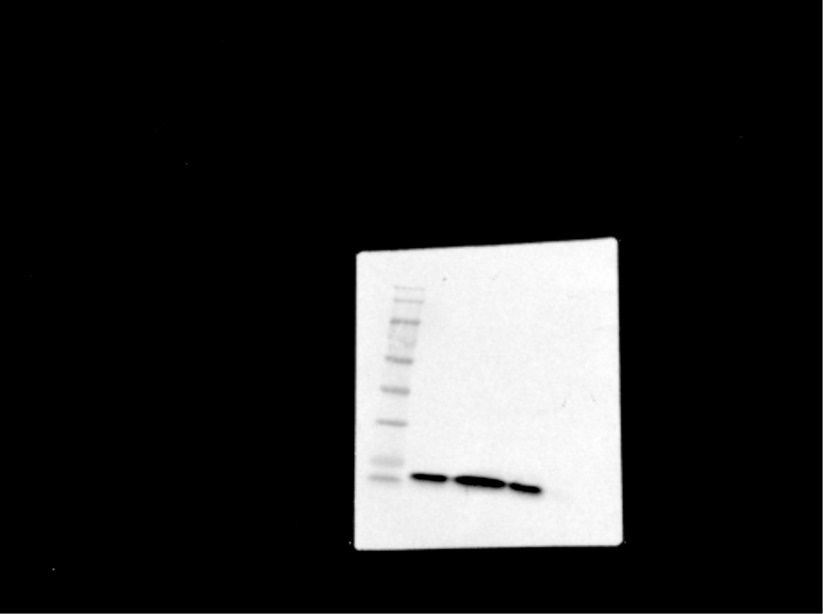


Figure 5J-1


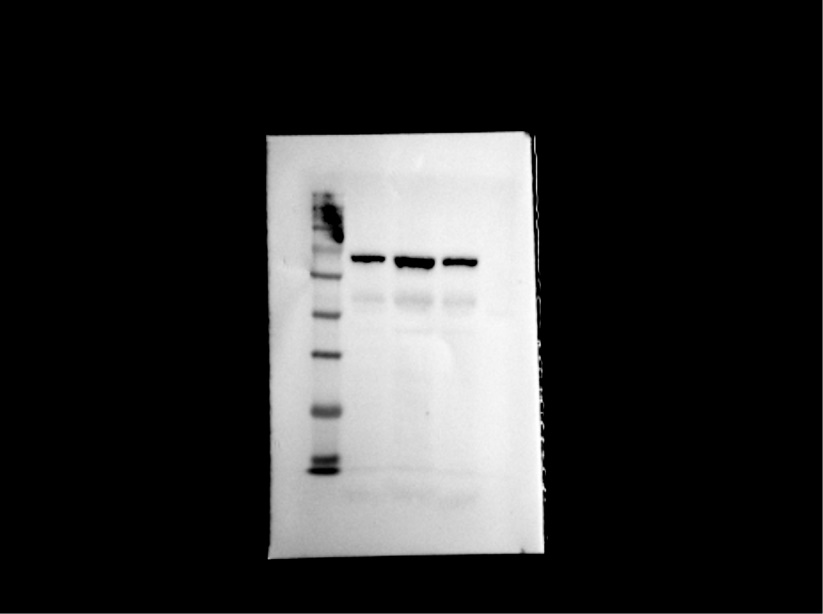


Figure 5J-2


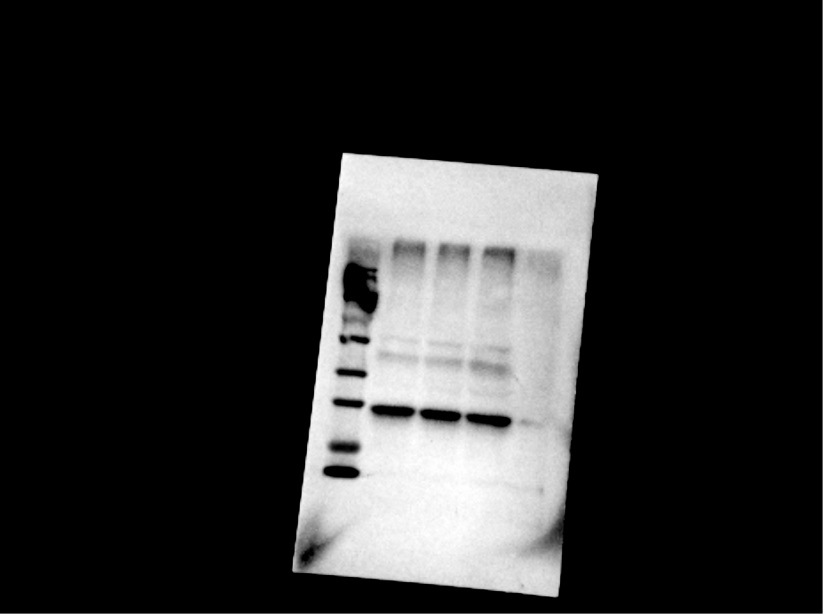


Figure 5J-3


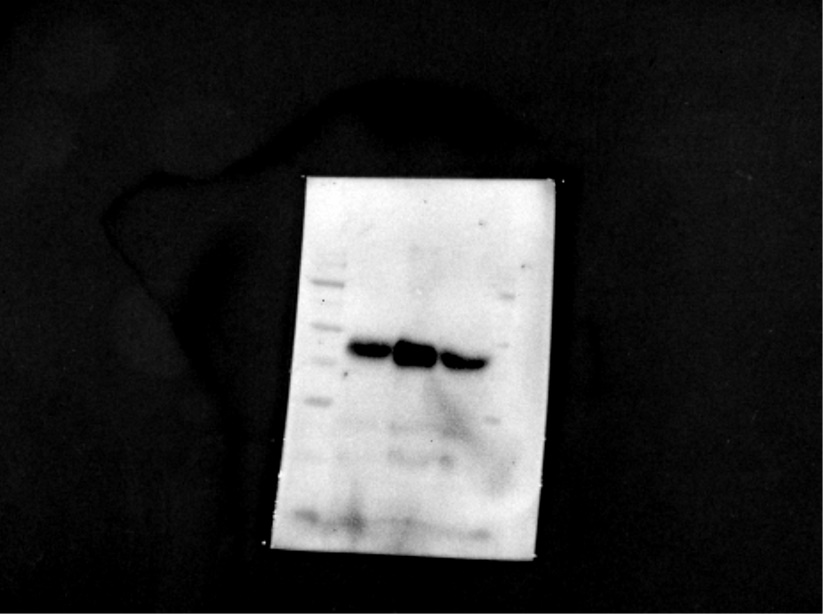


Figure 6D-1


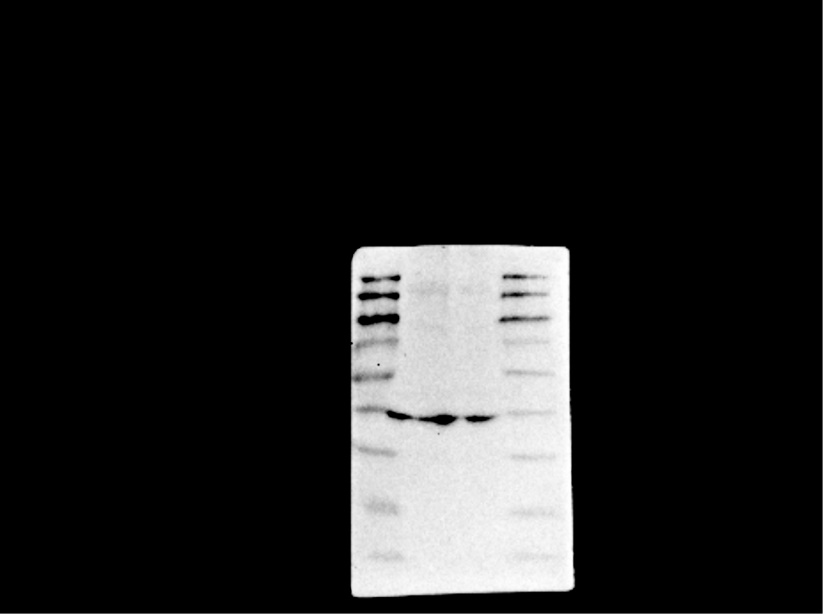


Figure 6D-2


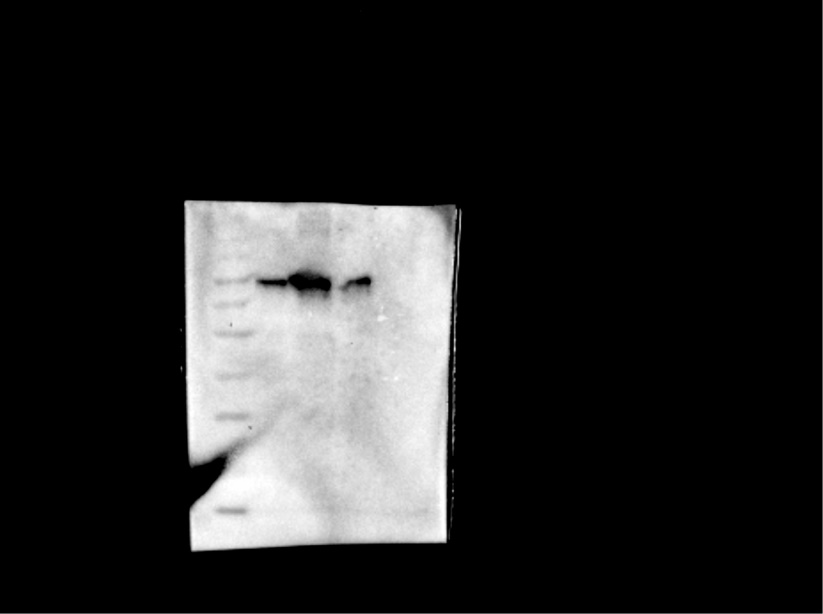


Figure 6D-3


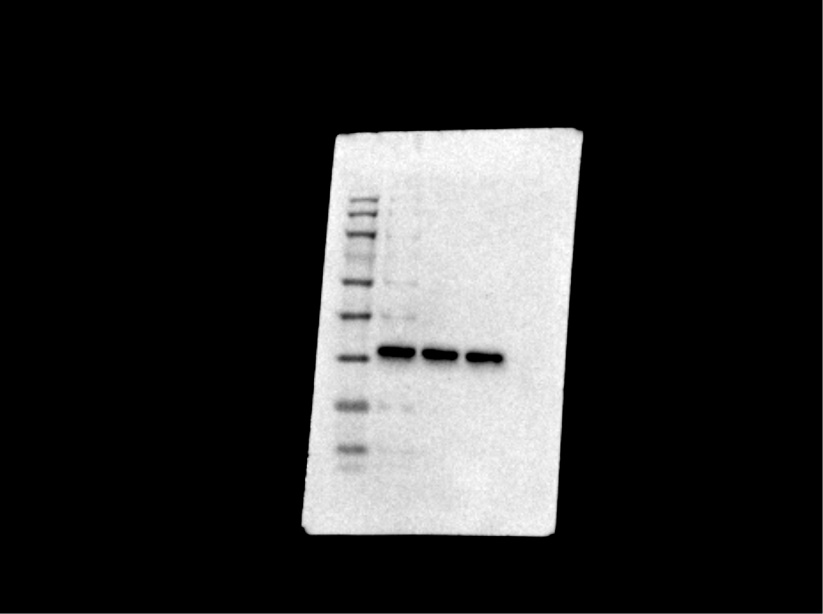


Figure 6D-4


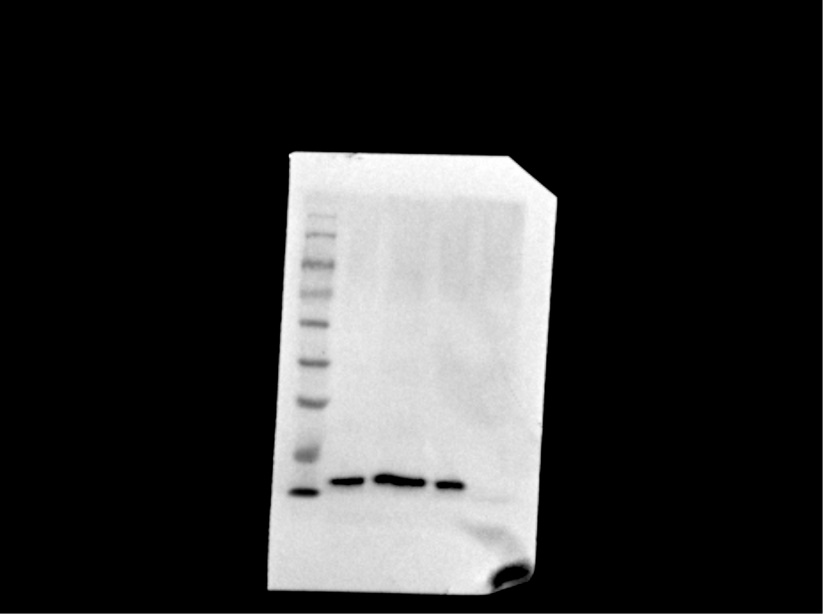


Figure 6D-5


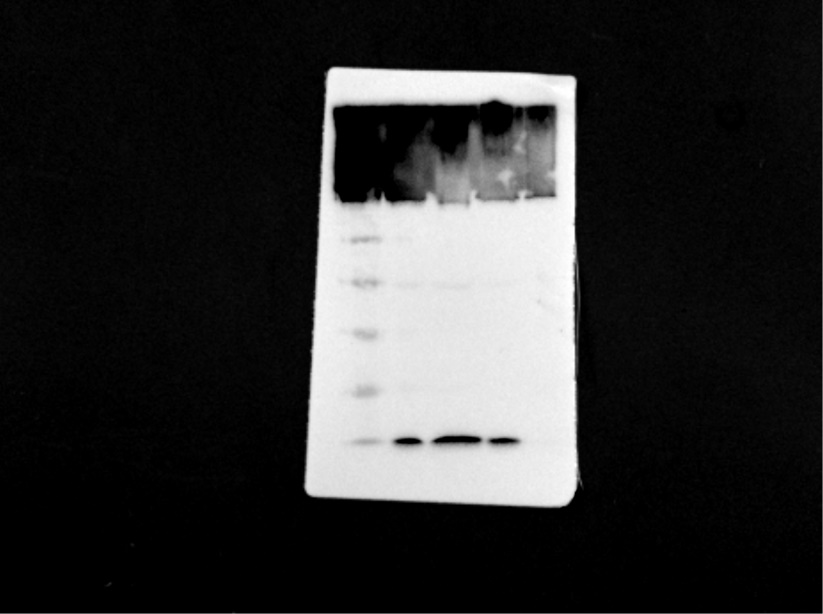


Figure 6D-6


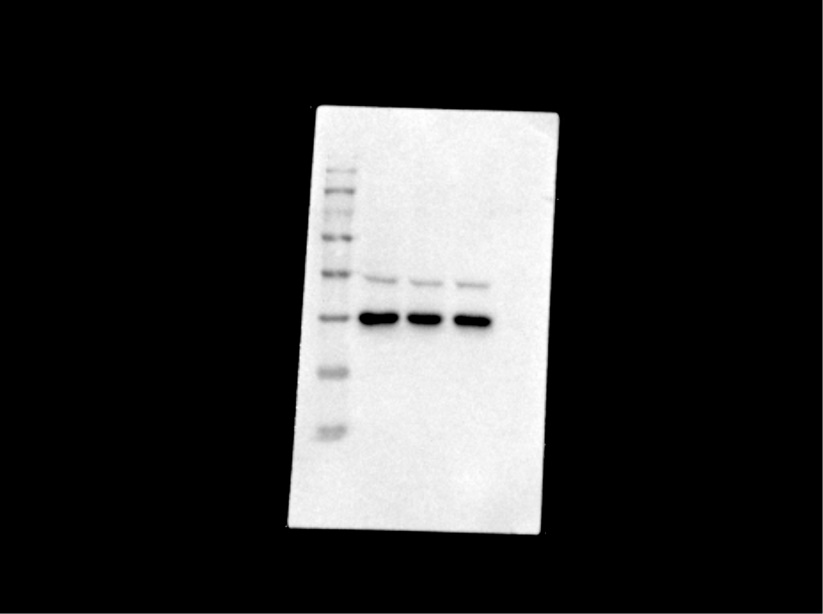


Figure 6D-7


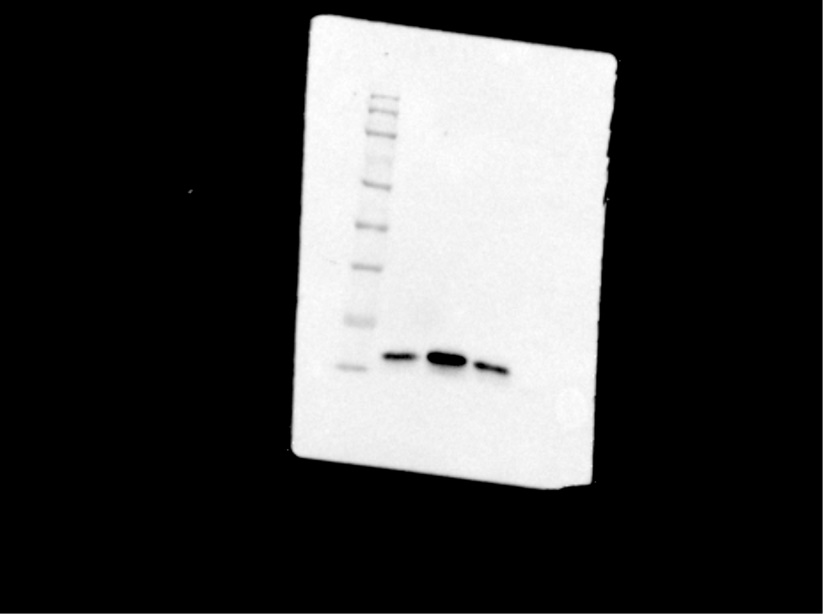


Figure 7N-1


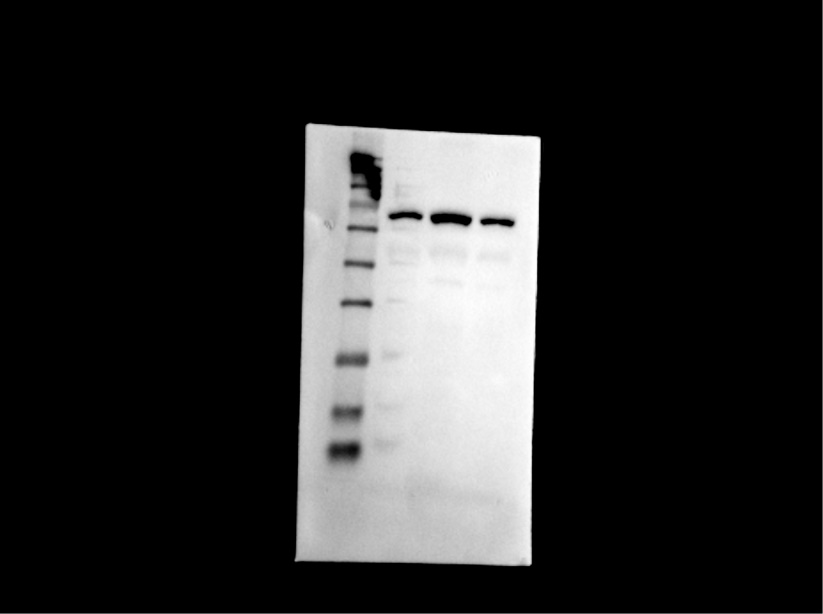


Figure 7N-2


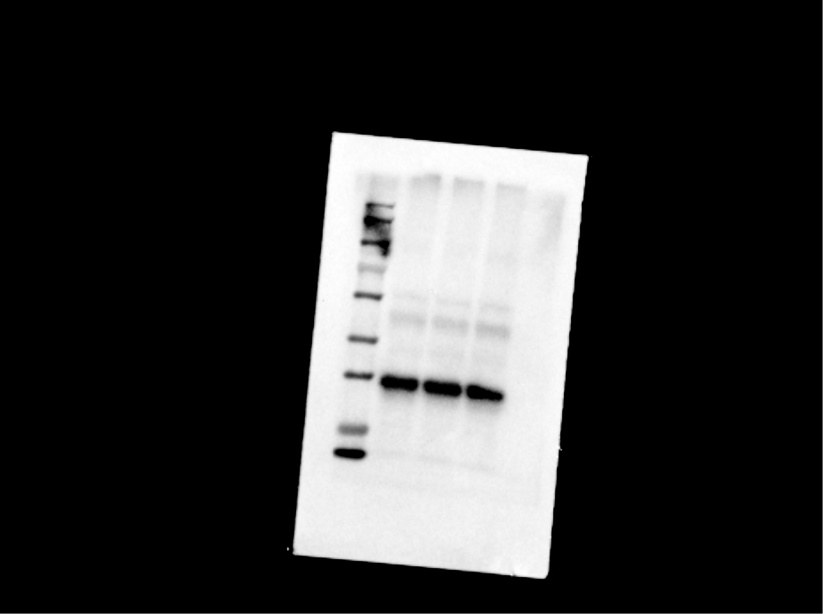


Figure 7N-3


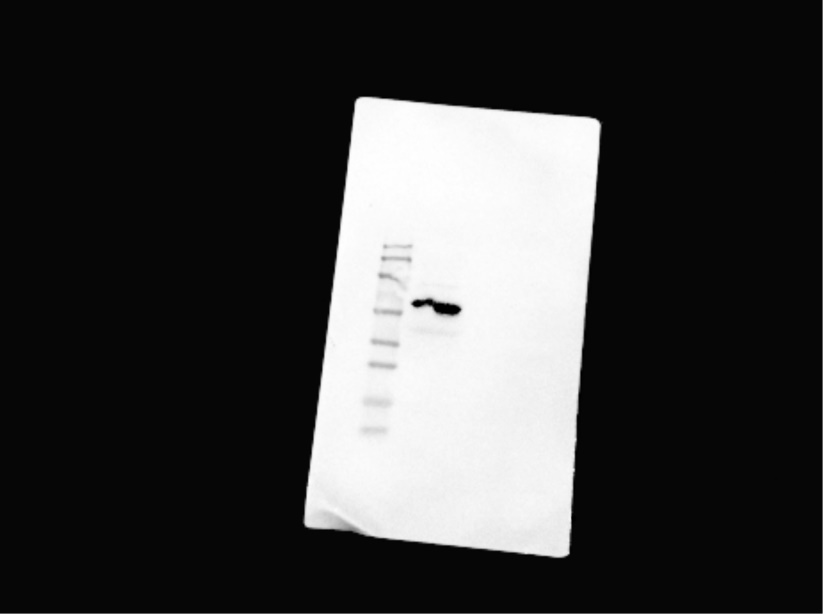


Figure S5-1


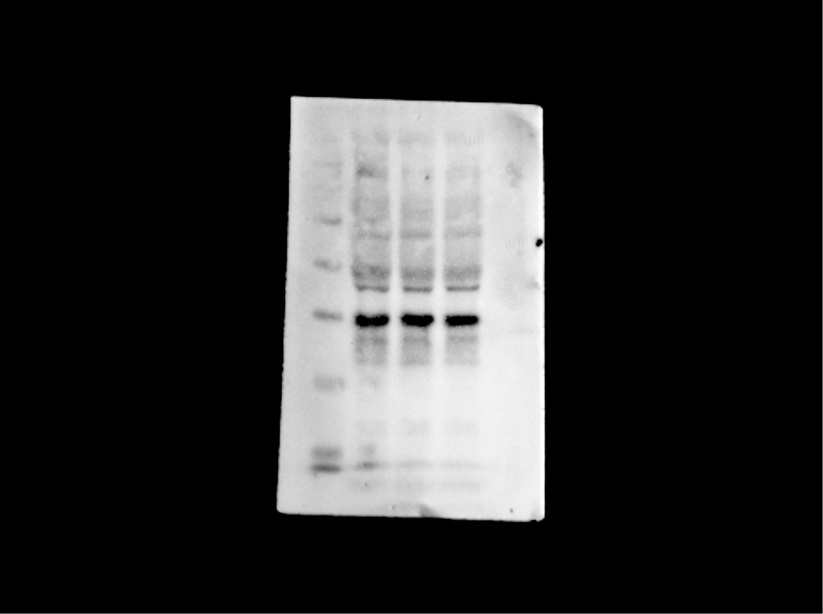


Figure S5-3


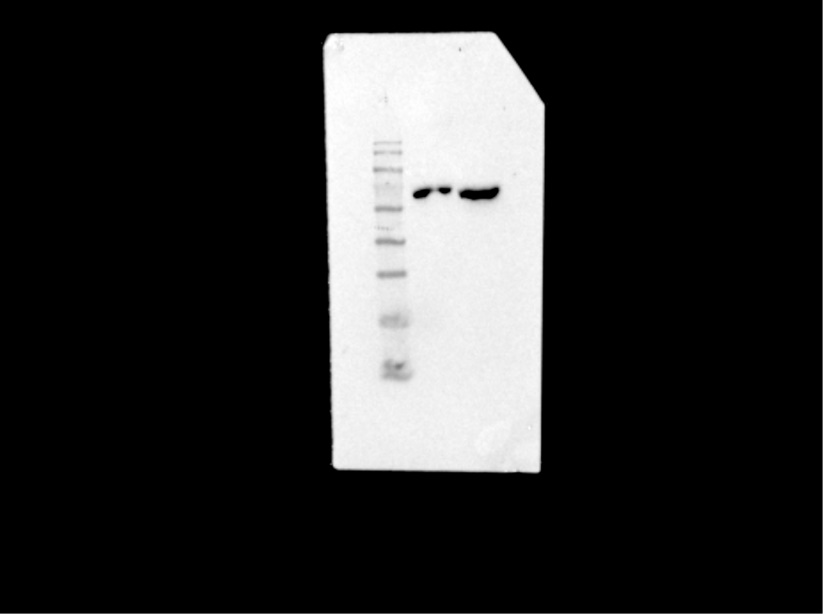


Figure S5F-1


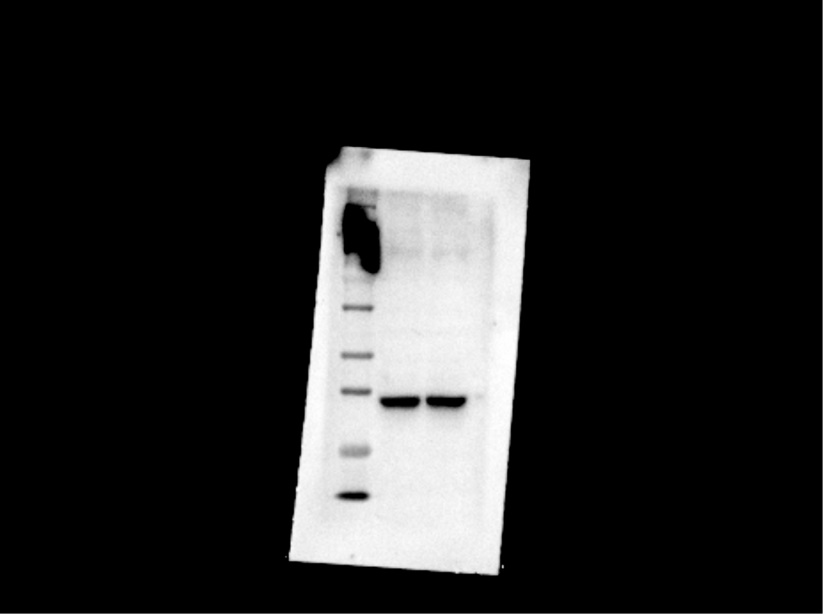


Figure S5F-2


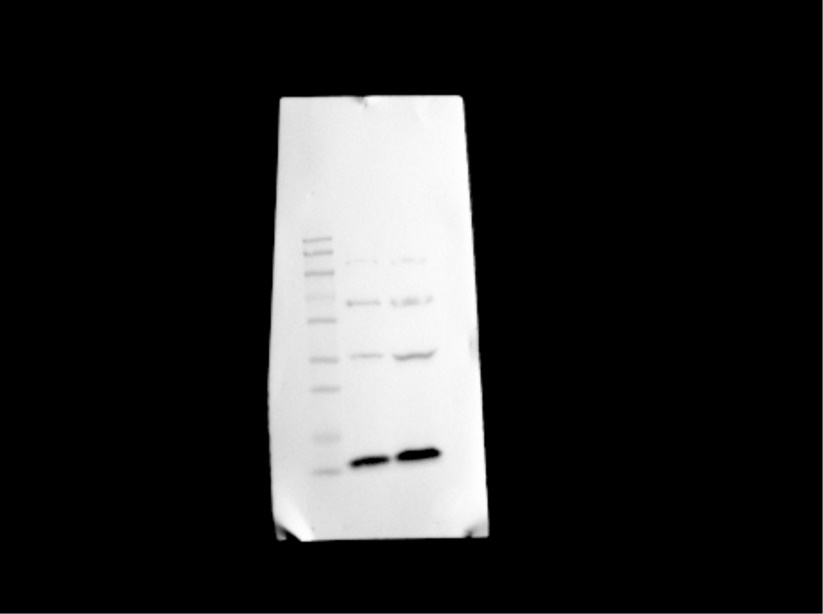


Figure S7J-1


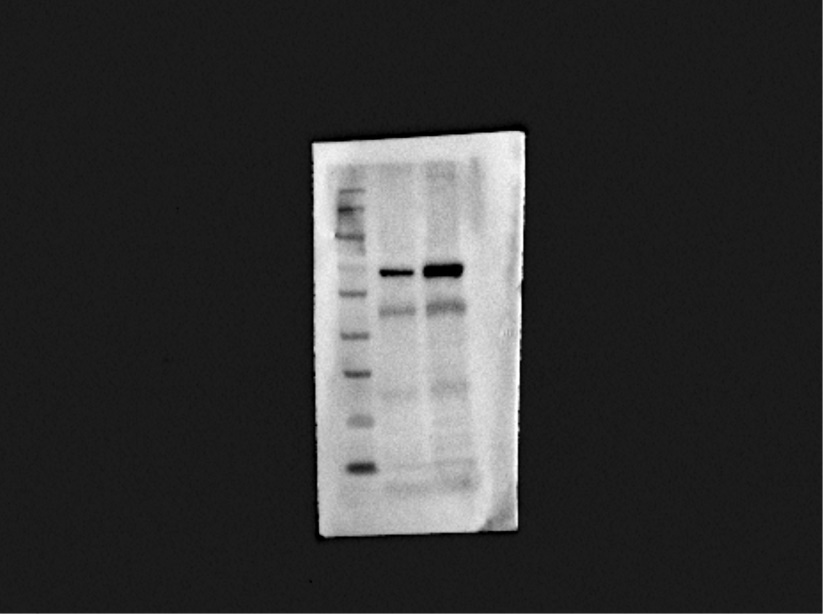


Figure S7J-2


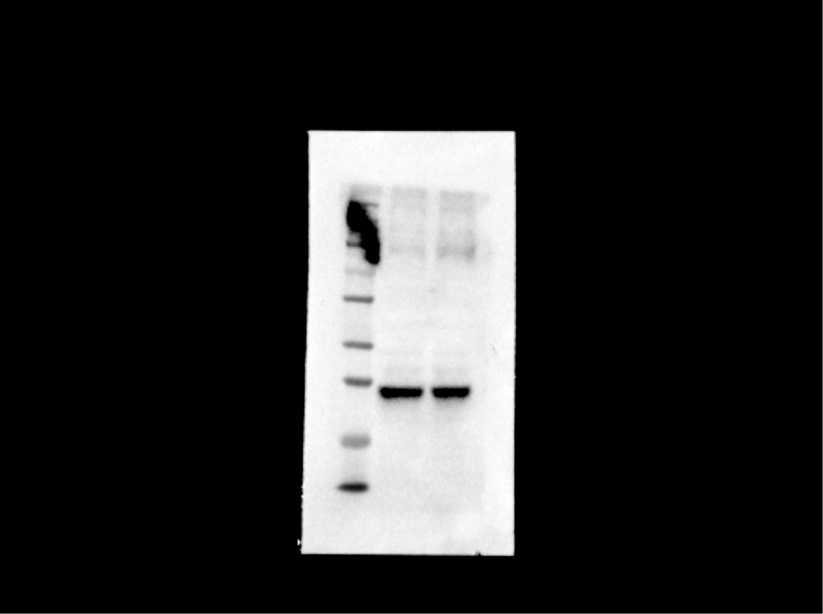


Figure S7J-3
