# Supplementary material for: Role of METTL16 in PPARγ methylation and osteogenic differentiation
Source: Cell Death Dis. 2025 Apr 10;16(1):271. doi: 10.1038/s41419-025-07527-x (PMC11986173; doi:10.1038/s41419-025-07527-x)
Supplement: Supplementary file 2 — Supplementary Tables and figures [file 41419_2025_7527_MOESM2_ESM.pdf]

Table S1. Cell types and marker genes

Cell Type	Marker genes
Neutrophils	FPR1, FCGR3B, ADGRG3
B cell	BLNK, CD74, CD37
Macrophage	CD14, CD163, CD68, AIF1
Erythrocyte	ALAS2, GYPA, HHB
T cell	CD27, CD3G, IL7R, SLAMF, CD6, ABLIM1
Monocyte	LYZ, FGL2
Bone Marrow Mesenchymal Stem Cells	CXCL12, COL1A2, ALPL, PDGFRA, ENG, NGFR, ITGB1, ACTA2, IL6ST

Table S2. Cell types and marker genes of Bone Marrow Mesenchymal Stem Cells

Cell Type	Markergenes
Osteoblasts	VCAN, ALPL, XIST
Chondrocytes	WIF1, OMD, NCAM1

Table S3. shRNA sequences

shRNAs	Sequence
sh- METTL16#1	5'- GATCTCATTAAAGTGGTGAAA -3'
sh- METTL16#2	5'- GCCAAGGAAACCCATTACTTT -3'
sh- METTL16#3	5'- GCACTTACGTACGTAACCAAA -3'
sh-PPARγ#1	5'- GCCTCCCTGATGAATAAAGAT -3'
sh-PPARγ#2	5'- GCTCCACACTATGAAGACATT -3'
sh-PPARγ#3	5'- GCCCTGGCAAAGCATTTGTAT -3'
sh-GPX4	5’- CTCATGAAGGTCTGCCTGAAA-3’
sh-NC	5'- TTAACTTTAATGAGTAGAC -3'

Table S4. RT-qPCR primer sequences

Gene	Primer Sequence
GAPDH (mouse)	F：5'- GGAGAGTGTTTCCTCGTCCC -3'
	R：5'- ATGAAGGGGTCGTTGATGGC -3'
METTL16 (mouse)	F：5'- GAGCGTAGCATCTGCGTTTC -3'
	R：5'- GCTTAGAGCCATCTCGCAGG -3'
PPARγ (mouse)	F：5'- CGCTGATGCACTGCCTATGA -3'
	R：5'- CGAGTGGTCTTCCATCACGG -3'
RUNX2 (mouse)	F：5'- GTGCGGTGCAAACTTTCTCCAG -3'
	R：5'- TGCTTGCAGCCTTAAATATTCCTG -3'
OSX / SP7 (mouse)	F：5'- ATGGCGTCCTCTCTGCTTG -3'
	R：5'- TGAAAGGTCAGCGTATGGCTT -3'
ALPL (mouse)	F：5'- GGGCCTGCTCTGTTTCTTCA -3'
	R：5'- CTGAGATTCGTCCCTCGCTG -3'
OPN / SPP1 (mouse)	F：5'- TGGCTGAATTCTGAGGGACTAAC -3'
	R：5'- TATAGGATCTGGGTGCAGGC -3'
OCN / BGLAP (mouse)	F：5'- GAACAGACAAGTCCCACACAG -3'
	R：5'- GAGCTGCTGTGACATCCATAC -3'
GPX4 (mouse)	F：5'- GCCAAAGTCCTAGGAAACGC -3'
	R：5'- CCGGGTTGAAAGGTTCAGGA -3'
SLC7A11 (mouse)	F：5'- AATACGGAGCCTTCCACGAG -3'
	R：5'- CTCCAGGGGCAGTCAGTTAG -3'

F：Forward，R：Reverse

Table S5. Details of the first antibody product

Name	Cat.	Dilution ratio	Manufacturer	Country	MW (kDa)
GAPDH	ab9485	1：2500	Abcam	UK	37
METTL16	ab252420	1：1000	Abcam	UK	64
PPARγ	ab272718	1：1000	Abcam	UK	57
RUNX2	ab236639	1：1000	Abcam	UK	57
Osterix	ab209484	1：1000	Abcam	UK	45
ALPL	MA5-24845	1：2000	Invitrogen	UK	80
Osteopontin	ab218237	1：1000	Abcam	UK	35
Osteocalcin	PA5-96529	1：1000	Invitrogen	UK	11
GPX4	ab125066	1：2000	Abcam	UK	22
SLC7A11	ab175186	1：2000	Abcam	UK	55

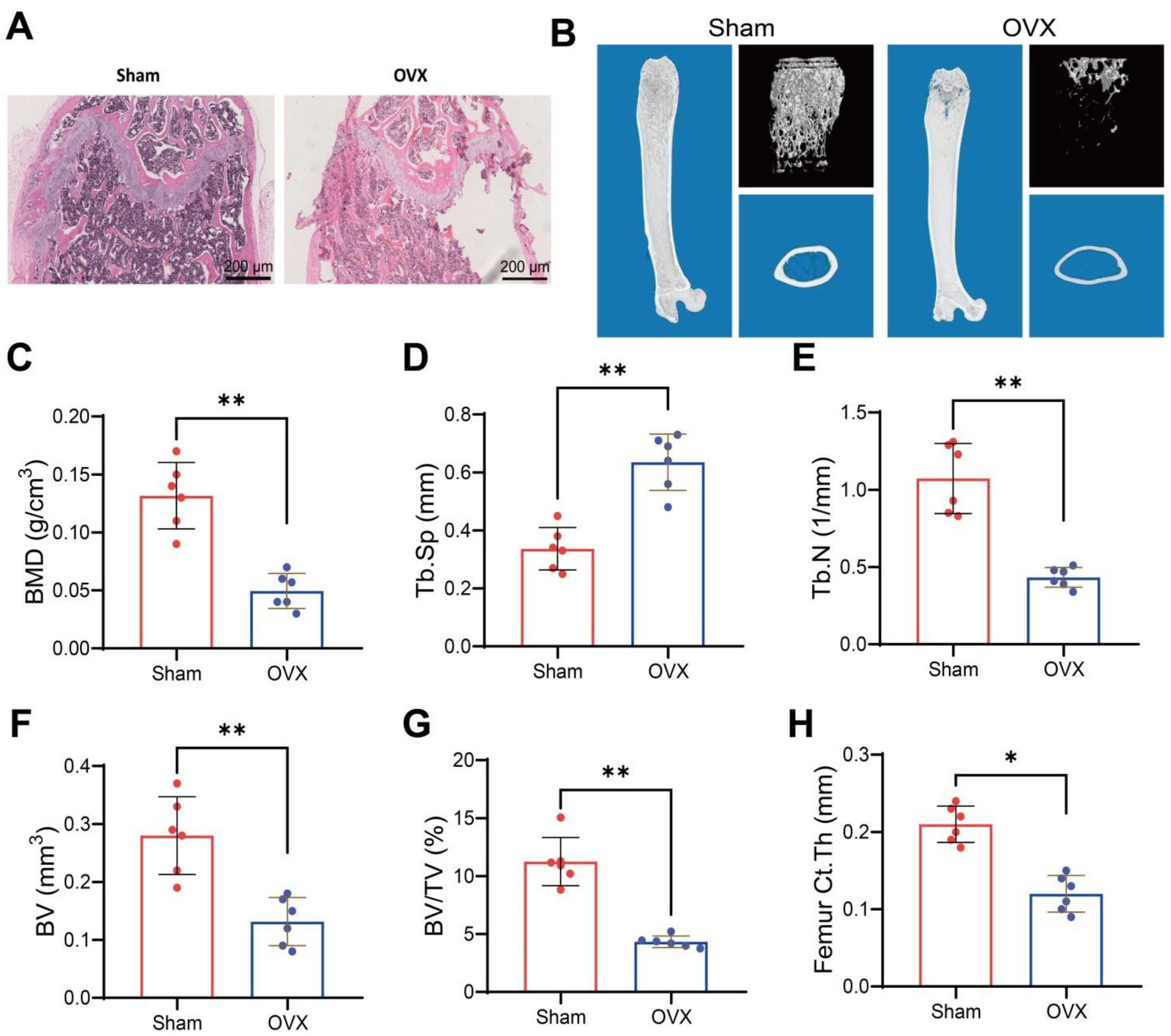

Figure S1. Validation of Osteoporotic Mouse Model Construction.

Note: (A) H&E staining of femoral mid-shaft in Sham and OVX group mice, scale bar: 200 μm. (B) Micro-CT images of Sham group and OVX group mice. (C-H) Micro-CT analysis and statistics of BMD, Tb.Sp, Tb.N, BV, BV/TV, and ct.Th in different groups of mice. Each group consisted of 6 mice. * indicates comparison between two groups, $p < 0.05$. ** indicates comparison between two groups, $p < 0.01$.

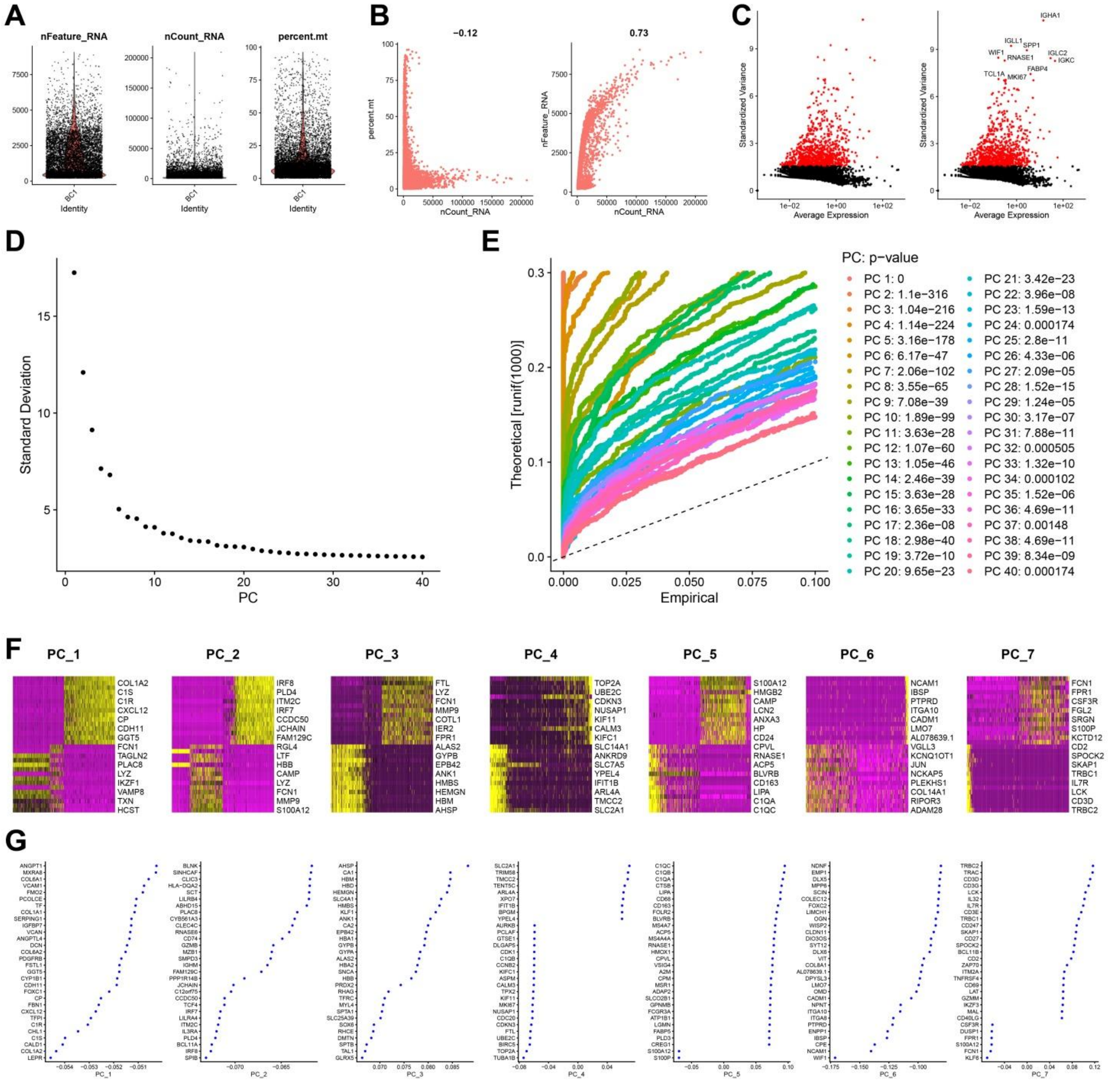

Figure S2. Quality control, filtering, and principal component analysis of scRNA-seq data.

Note: (A) Quality control for each cell in the scRNA-seq data, with three scatter plots displaying the number of nFeature_RNA, nCount_RNA, and percent. mt in each cell; (B) Scatter plots showing the correlation between filtered data, nCount_RNA, and percent. mt, as well as nCount_RNA and nFeature_RNA; (C) Variance analysis to select highly variable genes in the samples (red dots represent highly variable genes, black dots represent unchanged genes); (D) Distribution of standard deviation for PCs, with important PCs having larger standard deviations; (E) p-values of the top 40 PCs obtained from PCA analysis; (F-G) Heatmaps of feature genes and their expression levels in the top 7 PCs before PCA analysis, where yellow indicates upregulation and purple indicate

downregulation, n=1.

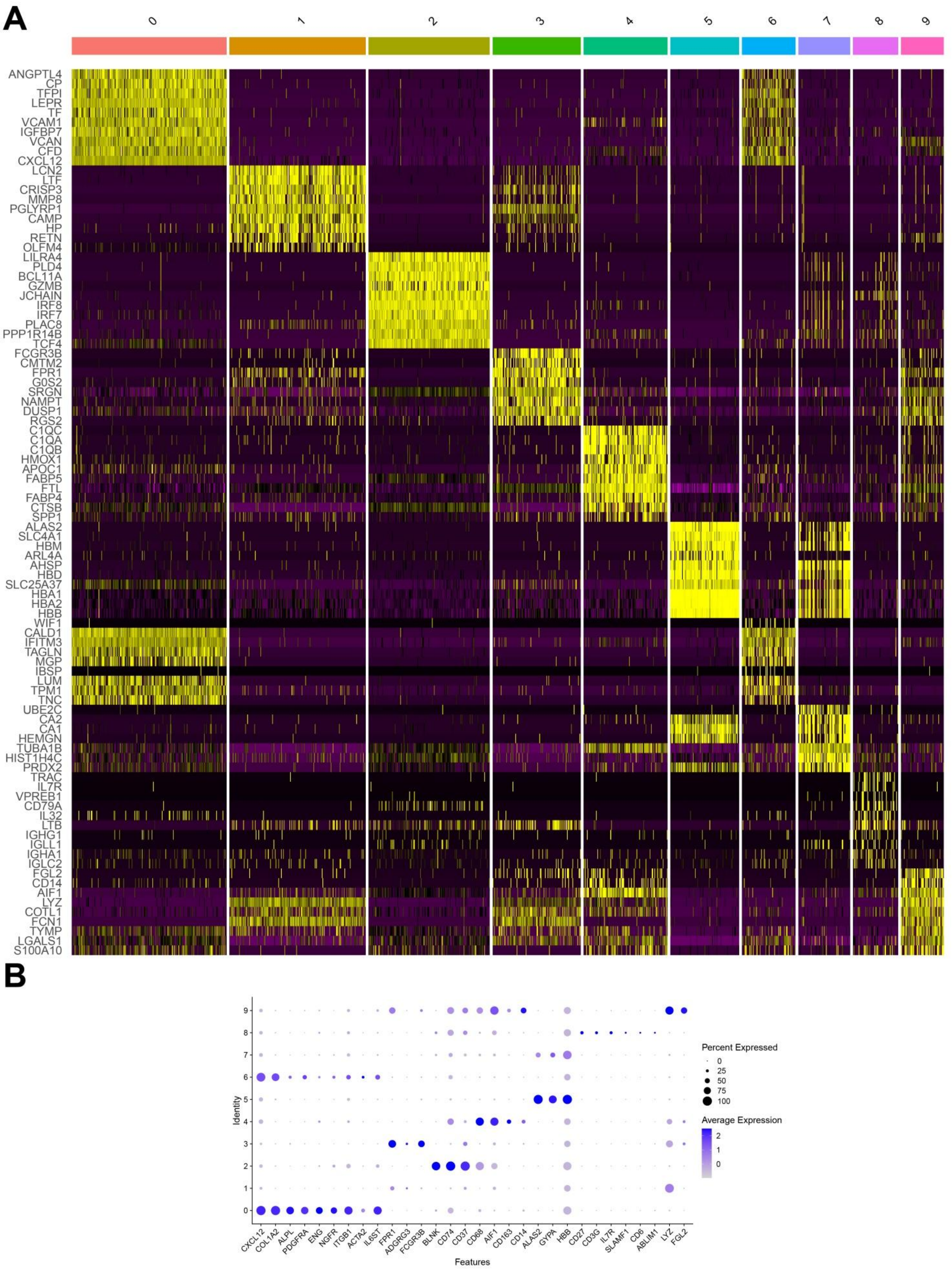

Figure S3. Marker Genes for scRNA Sequencing Cell Annotation.

Note: (A) Heatmap showing the top 10 differentially expressed genes in 10 cell clusters. (B) Dot

plot showing the expression levels of specific marker genes in 10 cell clusters.

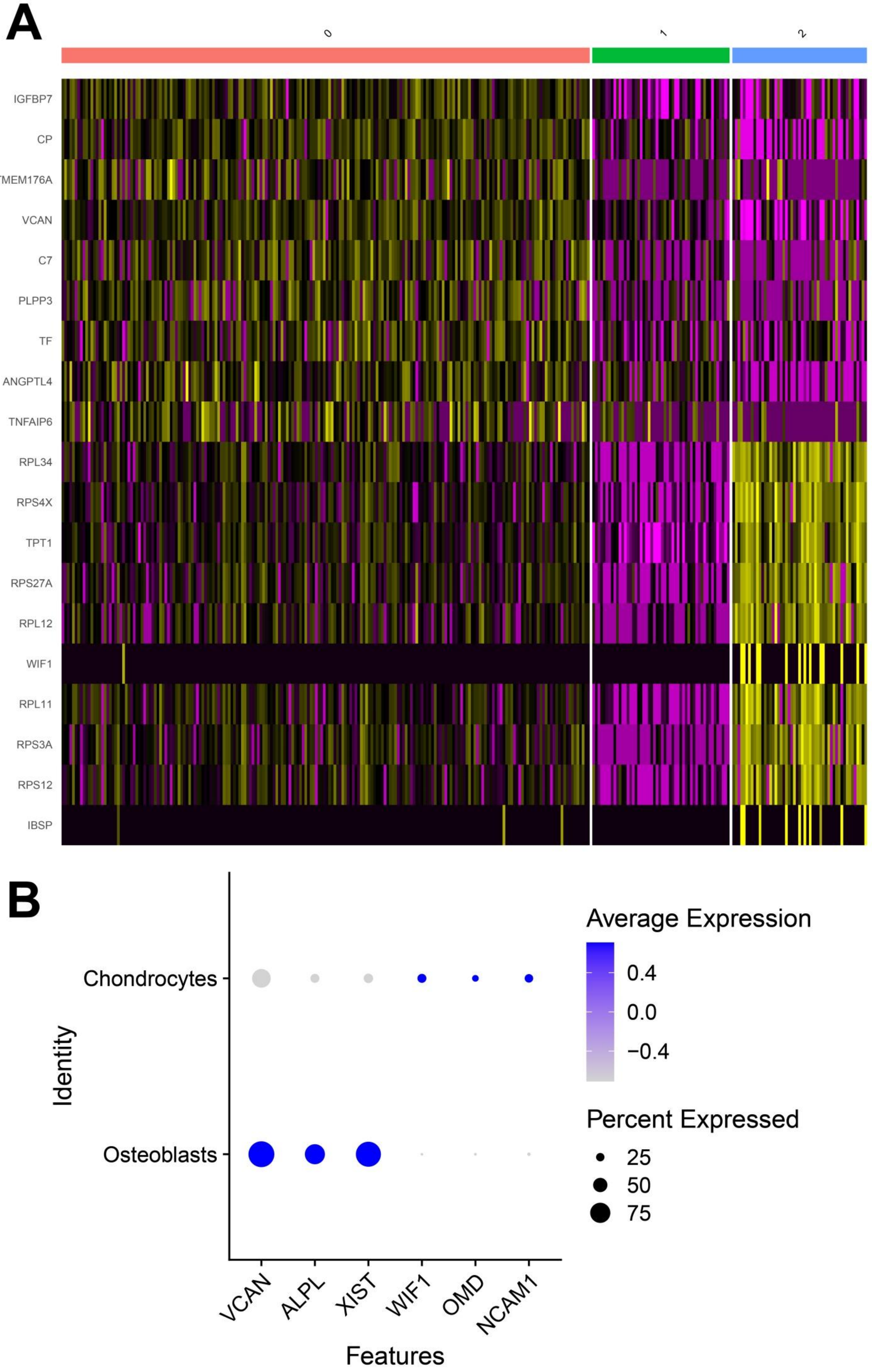
A
0
1
2
IGFBP7
CP
TMEM176A
VCAN
C7
PLPP3
TF
ANGPTL4
TNFAIP6
RPL34
RPS4X
TPT1
RPS27A
RPL12
WIF1
RPL11
RPS3A
RPS12
IBSP
B
Chondrocytes
Osteoblasts
Identity
VCAN
ALPL
XIST
WIF1
OMD
NCAM1
Features
Average Expression
0.4
0.0
−0.4
Percent Expressed
25
50
75

Figure S4. Differentially Expressed and Specific Marker Genes for Subgroup Analysis of BMSCs Clusters.

Note: (A) Heatmap showing differentially expressed genes in three cell clusters after subgroup analysis of BMSCs clusters. (B) Dot plot showing the expression levels of specific marker genes in BMSCs cluster subgroups.

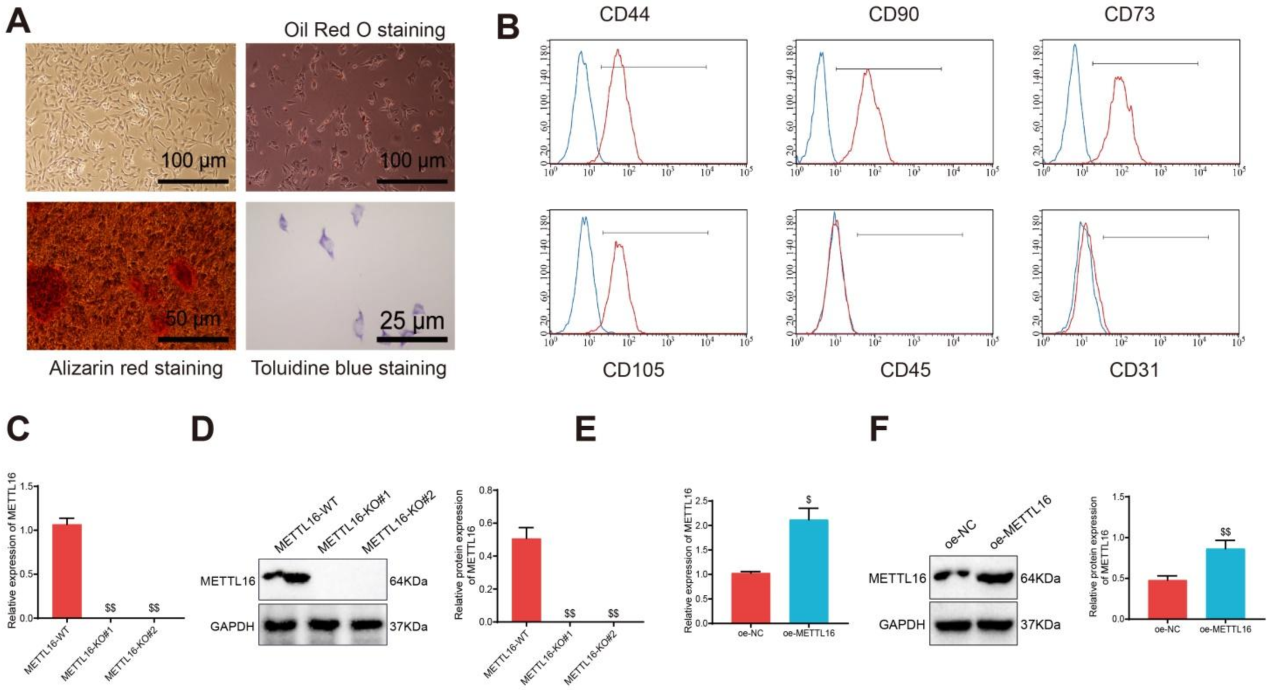

Figure S5. Identification of BMSCs and Transfection Results of METTL16 Knockout and Overexpression.

Note: (A) Observation of adipogenic, osteogenic, and chondrogenic differentiation abilities of BMSCs under a light microscope (Oil Red O Staining, scale bar: 100 μm; Alizarin red staining, scale bar: 50 μ m; Toluidine blue staining, scale bar: 25 μ m). (B) Flow cytometry analysis of CD31, CD44, CD45, CD73, CD90, and CD105 expression in BMSCs. (C) The performance of RT-qPCR for the measurement of expression levels of METTL16 in CRISPR/Cas9 gene-edited #1 and METTL16-KO#2 clones. (D) The conduction of Western blot analysis for the determination of expression levels of METTL16 in CRISPR/Cas9 gene-edited METTL16-KO#1 and METTL16-KO#2 clones. (E) RT-qPCR analysis of METTL16 expression in BMSCs after lentiviral transfection of METTL16 overexpression plasmid. (F) Western blot analysis of METTL16 expression in BMSCs after lentiviral transfection of METTL16 overexpression plasmid. $ indicates a difference compared to oe-NC group (p < 0.05). $$ indicates difference compared to METTL16-WT group (P < 0.05). Cell experiments were repeated 3 times.

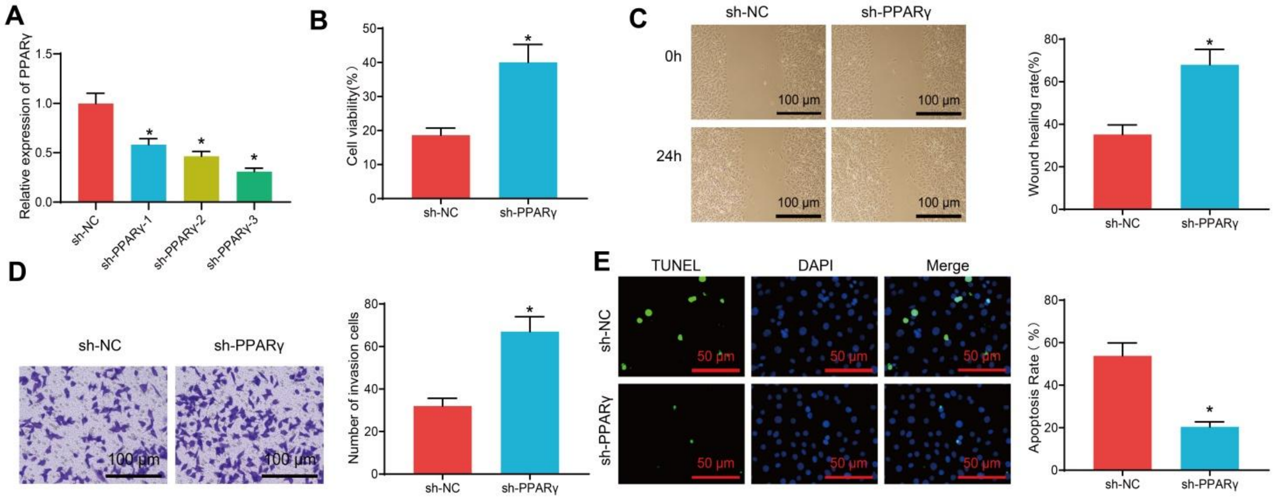

Figure S6. Impact of PPARγ on BMSCs Cellular Functions.

Note: (A) RT-qPCR analysis shows three PPARγ shRNA's silencing efficiency. (B) CCK-8 assay to measure BMSCs cell viability in different groups. (C) Scratch assay to assess the migration ability of BMSCs (scale bar: 100 μm). (D) Transwell assay to measure the migration and invasion ability of BMSCs (scale bar: 100 μm). (E) TUNEL staining to measure the apoptotic rate of BMSCs (Scale bar: 50 μm). * indicates a difference compared to sh-NC group (p < 0.05). Cell experiments were repeated 3 times.

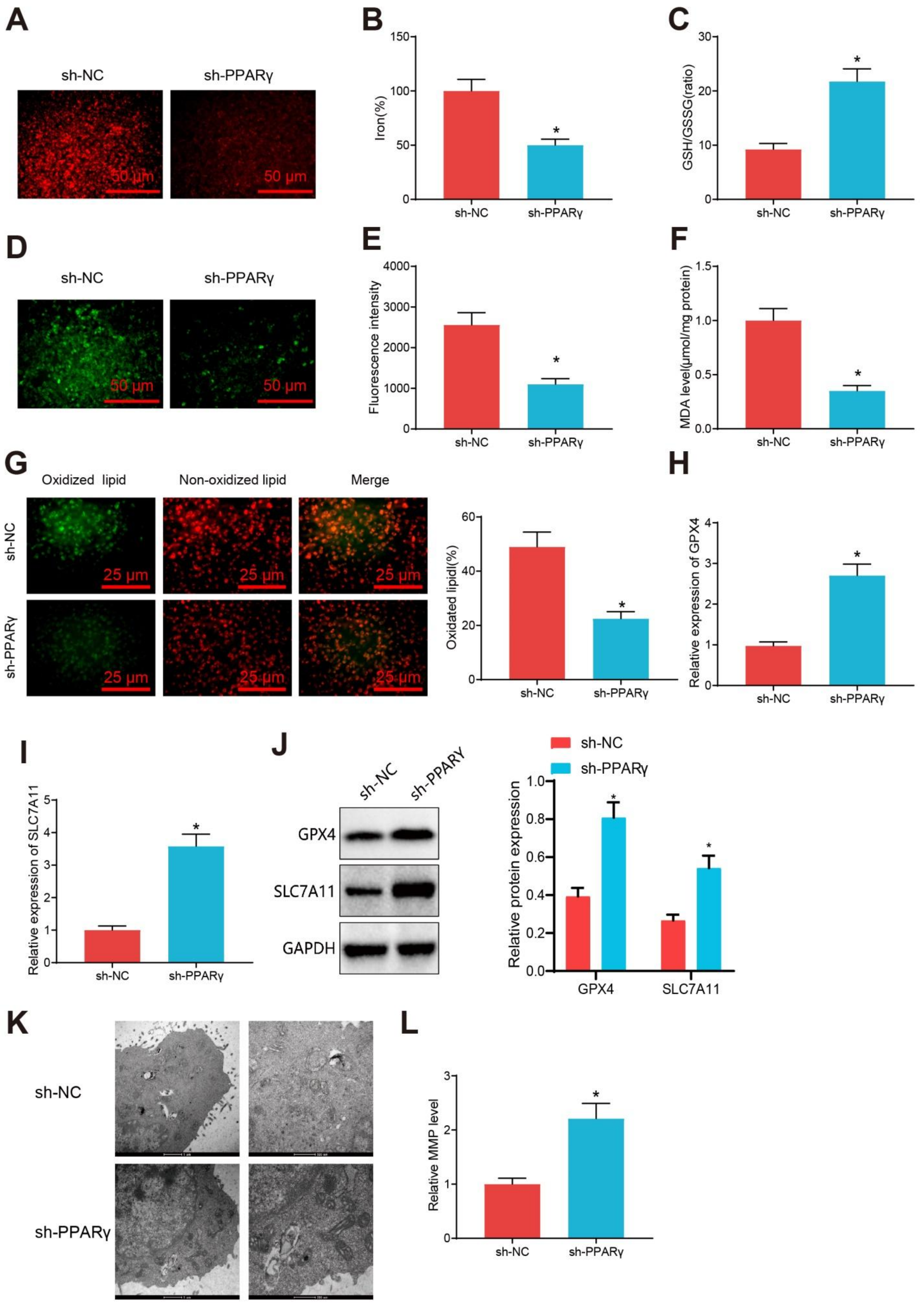

Figure S7. Effect of PPARγ knockdown on ferroptosis in BMSCs.

Note: (A) Representative fluorescence images of intracellular iron levels in BMSCs transfected with

different lentiviruses after 12 hours using FerroOrange staining (Scale bar=50 μm); (B) Statistical results of iron content in BMSCs of each group using a test kit; (C) GSH/GSSG ratio in BMSCs after different treatments; (D-E) Visualization of ROS production in BMSCs using DCFH-DA and statistical analysis by flow cytometry, Scale bar=50 μm; (F) Detection of MDA content in each group; (G) Representative confocal images of BMSCs stained with C11-BODIPY 581/591. Red indicates non-oxidized lipids, green represents oxidized lipids (Scale bar=25 μm); (H-I) Transcriptional levels of ferroptosis-related genes SLC7A11 and GPX4 detected by RT-qPCR; (J) Western blot analysis of the protein expression levels of ferroptosis-related genes SLC7A11 and GPX4 in BMSCs from each group. (K) Observation of mitochondrial morphology in cells by transmission electron microscopy, Scale bar=1 μm; (L) Detection of mitochondrial membrane potential (MMP) in each group of cells using JC-1. * indicates $p < 0.05$ compared to the sh-NC group; cell experiments were repeated three times.

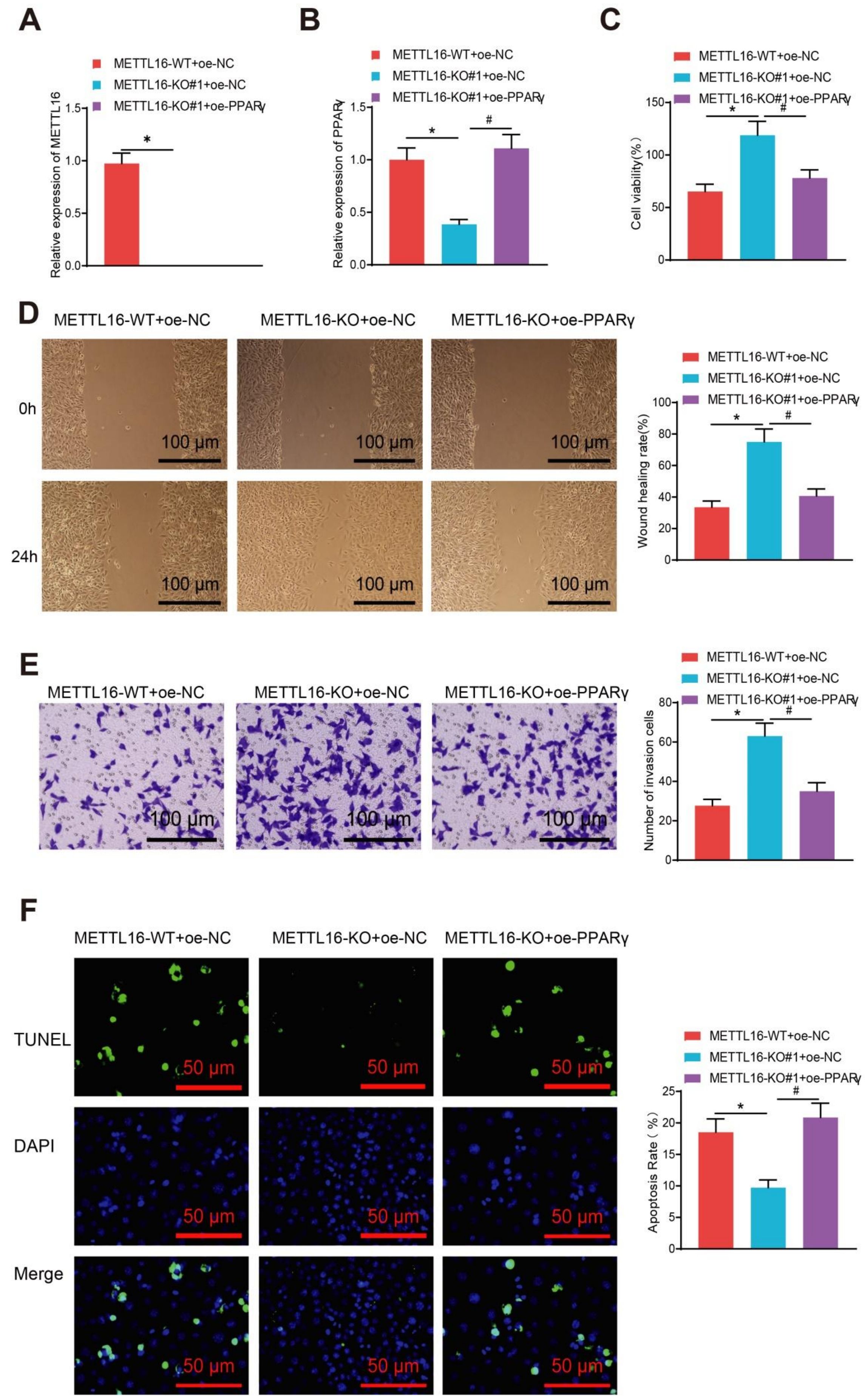

Figure S8. Impact of the METTL16/PPARγ Axis on BMSCs Cellular Functions.

Note: (A, B) RT-qPCR analysis of METTL16 and PPARγ mRNA expression levels in different groups of BMSCs. (C) CCK-8 assay to measure BMSCs cell viability in different groups. (D) Scratch assay to assess the migration ability of BMSCs (scale bar: 100 μm). (E) Transwell assay to measure the migration and invasion ability of BMSCs (scale bar: 100 μm). (F) TUNEL staining to measure the apoptotic rate of BMSCs (Scale bar: 50 μm). * indicates difference compared to METTL16-WT + oe-NC group ($p < 0.05$). # indicates difference compared to METTL16-KO + oe-NC group ($p < 0.05$). Cell experiments were repeated 3 times.
